# Non-vitamin K Antagonist Oral Anticoagulants and Cognitive Impairment in Atrial Fibrillation: Insights From the Meta-Analysis of Over 90,000 Patients of Randomized Controlled Trials and Real-World Studies

**DOI:** 10.3389/fnagi.2018.00258

**Published:** 2018-10-02

**Authors:** Chi Zhang, Zhi-Chun Gu, Long Shen, Mang-Mang Pan, Yi-Dan Yan, Jun Pu, Xiao-Yan Liu, Hou-Wen Lin

**Affiliations:** ^1^Department of Pharmacy, Renji Hospital, School of Medicine, Shanghai Jiaotong University, Shanghai, China; ^2^Department of Cardiology, Renji Hospital, School of Medicine, Shanghai Jiaotong University, Shanghai, China

**Keywords:** atrial fibrillation, cognitive impairment, non-vitamin K antagonist oral anticoagulants, warfarin, real-world study, meta-analysis

## Abstract

**Background:** The relationship between the use of non-vitamin K antagonist oral anticoagulants (NOACs) and the impairment of cognition in atrial fibrillation (AF) remains unknown.

**Methods:** A comprehensive database search of Medline, Embase, Cochrane Library databases, and ClinicalTrials.gov Website was performed for randomized controlled trials (RCTs) reporting cognitive impairment events and observational nationwide database studies reporting adjusted hazard ratio (HR) in AF patients with NOACs. The primacy outcome was a composite of any cognitive impairment. Summary of HRs and 95% confidence intervals (95%CI) were calculated using the fixed- and random-effects models. Subgroup analyses were undertaken according to the individual NOACs, study types, and duration of follow-up.

**Results:** Finally, eight studies including 97,595 patients (77,643 patients in 6 RCTs and 19,952 patients in 2 observational database studies) met the inclusion criteria, among which 55,337 (56.7%) patients were receiving NOACs and 42,258 (43.3%) patients were receiving vitamin K Antagonists (VKAs) or acetylsalicylic acid. The results showed a borderline significant association between the use of NOACs and the lower risk of cognitive impairment when compared with VKAs/ acetylsalicylic acid (HR: 0.80, 95%CI: 0.63–0.98 for fixed-effects model; HR: 0.77; 95%CI: 0.53–1.01 for random-effects model), with no significant heterogeneity between the studies (*I*^2^ = 39.4%, *P* = 0.12). The results were consistent across the key subgroups (*P*_interaction_ > 0.05 for each).

**Conclusions:** The results indicated that the use of NOACs might lower the tendency on the risk of cognitive impairment in comparison to VKAs/acetylsalicylic acid, and further RCTs and real-world studies are required on an urgent basis to obtain a robust result.

## Introduction

Atrial fibrillation (AF) and cognitive impairment, which predominantly affect the elderly, are expected to be among the most prominent global epidemiological trends in the twenty-first century, thus bringing an overwhelming burden to the health care system worldwide (Dietzel et al., 2018). These two diseases share many common risk factors (hypertension, diabetes mellitus, vascular disease, and heart failure), and growing evidences suggest that AF is strongly associated with the increased risk of cognitive dysfunction and dementia (Jacobs et al., 2017; Dagres et al., 2018; Pastori et al., 2018). It is reported that patients with AF are twice as likely to develop dementia compared to those without AF (Jacobs et al., 2017). The potential relationship between AF and cognitive impairment is not fully illuminated. AF-related ischemic stroke results from static blood produced by fibrillation of atrium, leading to thrombus formation and embolism to the brain (Kamel et al., 2016). So far, it is widely accepted that ischemic stroke, either overt or silent stroke, is one of the important causes leading to cognitive decline (Hui et al., 2015; Dietzel et al., 2018). In addition, the presence of cerebral hypoperfusion, chronic inflammation, or endothelial dysfunction is also involved in AF-related cognitive impairment (Jacobs et al., 2017).

Stroke prevention therapy, particularly oral anticoagulation, is the principal priority in the management of AF and is strongly recommended by the current guidelines (Steffel et al., 2018). Nevertheless, there is still a paucity of evidence regarding the association between effective anticoagulation and the risk of cognitive impairment. One recent study showed that warfarin-treated AF patients with a low percentage of time in the therapeutic range (TTR) were at a high risk of dementia due to under- or over-anticoagulation (Jacobs et al., 2014). Another observational data suggested that the delays in warfarin therapy could increase the risk of dementia in AF patients without a dementia history (Bunch et al., 2017). Accordingly, it is plausible to assume that warfarin might contribute to the preserved cognitive function owing to the prevention of stroke. Non-vitamin K antagonist oral anticoagulants (dabigatran, rivaroxaban, apixaban, edoxaban, and betrixaban), targeting either thrombin or factor Xa, are therapeutically advantageous over or at least non-inferior in terms of thromboprophylaxis, and have the lower intracranial hemorrhage and micro-hemorrhage rates in comparison to warfarin, thus being the class I recommendation for stroke prevention in non-valvular atrial fibrillation (NVAF) patients (Husted et al., 2014; Saito et al., 2015; Kirchhof et al., 2017). Moreover, the NOACs could offer substantially lower variability in therapeutic anticoagulation effect, which leads to the hypothesis that the NOACs might have a better protection against AF-related cognitive dysfunction than warfarin. Regretfully, limited information from randomized controlled trials (RCTs) have confirmed this hypothesis. When evidences from RCTs were insufficient to obtain conclusion, well-designed real-world studies could provide more valuable information. Therefore, the aim of the present study is to summarize the current evidences including the available RCTs and high-quality real-world studies for carrying out a rigorous meta-analysis in terms of potential association between NOACs and cognitive function in AF, as well as to try to confirm this hypothesis.

## Methods

### Data sources and searches

The present study was conducted in accordance with the standards outlined in the Cochrane Handbook and the PRISMA Statement for Reporting Systemic Reviews and was prospectively registered in PROSPERO (registration no. CRD42018103849) (Moher et al., 2009; Higgins, 2011; Gu et al., 2018). Databases of Medline, Embase, and Cochrane Library were searched to identify all the potentially eligible studies from inception to April 23rd, 2018 with the following searching strategy: “dabigatran” or “Pradaxa” or “rivaroxaban” or “Xarelto” or “apixaban” or “Eliquis” or “edoxaban” or “Savaysa” or “betrixaban” or “Bevyxxa” or “Non-vitamin K antagonist oral anticoagulants” or “NOACs” or “direct oral anticoagulants” or “DOACs” or “novel oral anticoagulants” or “new oral anticoagulants” or “factor Xa inhibitors” or “factor II a inhibitors” in combination with “atrial fibrillation” or “AF.” In addition, unpublished trials were identified from the website, ClinicalTrials.gov. Reference of identified records and relevant reviews were manually checked and then further scrutinized to identify any potentially eligible articles. Two of the authors of the article who reviewed the content (Chi Zhang and Zhi-Chun Gu) independently searched the databases, and all the disagreements were resolved by consulting a third author (Xiao-Yan Liu).

### Study selection and outcomes

To be eligible for inclusion, the design of the studies had to be RCTs or observational studies of NOACs that reported outcomes of cognitive function. For observational studies, only the high-quality nationwide or health insurance database studies that reported adjusted or matched results were eligible. For outcome analyses in which the studies have applied the same sources of data, only the study with the longest period was included unless the study period did not overlap between studies or unless the study included data from another source. Studies that were published only in the abstract form or did not report matched or adjusted results were excluded. The primacy outcome was cognitive impairment, defined as a composite of any cognitive dysfunction. The secondary outcomes included narrow definition of cognitive impairment (including dementia, dementia Alzheimer's type, vascular dementia, senile dementia, frontotemporal dementia, and dementia with Lewy bodies) and individual cognitive impairment reported in the study. Two of the authors of the article who reviewed the content (Chi Zhang and Zhi-Chun Gu) independently assessed all the study titles and abstracts to determine the eligibility, and thereafter the entire paper was retrieved and assessed on the basis of inclusion criteria. All the discrepancies were resolved by consulting a third author (Xiao-Yan Liu).

### Data extraction, quality evaluation, and bias assessment

Prespecified data variables were extracted independently by two of the authors of the article who reviewed the content (Chi Zhang and Zhi-Chun Gu), including the study characteristics, patient demographics, clinical characteristics, and data of reported cognitive function. The detailed data of RCTs of cognitive function that was not reported in the original publications was further extracted from the website, ClinicalTrials.gov. As cognitive impairment can be represented in various forms, and for a purpose of meaningful analysis, the following outcomes were used as cognitive impairment, which included amnesia, cognitive disorder, dementia, dementia Alzheimer's type, global amnesia, memory impairment, Parkinson's disease, Parkinsonism, vascular dementia, senile dementia, sensory disturbance, frontotemporal dementia, altered state of consciousness, amnestic disorder, and dementia with Lewy bodies. The methodological quality of the included RCTs was evaluated based on the Cochrane Collaboration Risk of Bias Tool (2011; Wei et al., 2016). Since observational studies have a higher risk of bias compared to RCTs, we did not use the quality assessment tools designed for the RCTs. Instead, the risk of bias was considered in the observational study design and methods that were used to mitigate certain bias, and was assigned to the following domains: use of matched or adjusted method to handle selection bias; possibility for residual confounding; use of methods to deal with time-varying covariates and information censoring; detailed reporting of baseline characteristics and outcome measures (Romanelli et al., 2016). The potential publication bias was explored by visual inspection of funnel plots (Duval and Tweedie, 2000; Gu et al., 2017).

### Data analysis

Hazard ratios (HRs) and 95% confidence intervals (CIs), according to various forms of cognitive impairment, were calculated using the fixed- and random-effects models. For a composite outcome, the individual data of cognitive impairment were merged as one camp, the corresponding HRs and CIs were first calculated for the included RCTs, and then those included observational studies that reported the adjusted HRs and 95%CI were pooled based on the fixed- and random-effects models. Statistical heterogeneity was assessed with *I*^2^ test and Q statistic. The *I*^2^ values of >50% represented considerable heterogeneity, and a *p*-value of <0.05 at Q statistic was considered as significant heterogeneity (Higgins and Thompson, 2002). Subgroup analyses were conducted according to the individual NOACs (dabigatran, rivaroxaban, apixaban, and edoxaban), study types (RCTs and database studies), and duration of follow-up (>1 year or <1 year). For exploring the potential effect modifiers on the risk of cognitive impairment, a meta-regression analysis was performed to test the demographic characteristics of the included studies. Sensitivity analyses were carried out to assess the robustness of results with the sequential elimination of the individual studies. In addition, further analyses were conducted to identify the effect by excluding the studies that involved catheter ablation, acetylsalicylic acid as control and low dosage arms of NOACs, or by adding the data of magnetic resonance imaging (MRI) sub-study in AXAFA-AFNET 5 trial. All statistical analyses were performed by using the STATA software (version13, Statacorp, College Station, Texas, USA), and a *P-*value of <0.05 indicated a statistically significant difference.

## Results

### Study evaluation

The process of the literature search and inclusion is shown in Figure S1, Tables S1, S2. Eventually, 6 RCTs and 2 observational studies were included (Connolly et al., 2009, 2011; Granger et al., 2011; Patel et al., 2011; Giugliano et al., 2013; Jacobs et al., 2016; Friberg and Rosenqvist, 2018; Kirchhof et al., 2018). A total of 97,595 patients, consisting of 55,337 patients with NOACs and 42,258 patients with vitamin K Antagonists (VKAs) or acetylsalicylic acid, were included. The characteristics of the 6 RCTs are outlined in Table 1. The publication periods ranged from 2009 to 2018, with the up-to-date AXAFA-AFNET 5 study published in March 2018. The follow-up duration ranged widely from 0.25 to 2.8 years. The included RCTs contained 45,361 patients receiving NOACs and 32,282 patients receiving VKAs or acetylsalicylic acid. The characteristics of the 2 database studies are outlined in Table 2, consisting of 9,976 patients with dabigatran treatment and 9,976 patients with warfarin treatment. The 2 database studies applied the propensity score matching method to minimize the influence of confounding factors and heterogeneity of patient characteristics between comparison groups. As shown in Table 3, patient demographics of the included studies were similar across eight studies, but the clinical characteristics were different in terms of risk factors of stroke. The included studies satisfied all bias tool items except for the RE-LY and AXAFA-AFNET 5, which were not blinded. Thus, the included studies were of modest to high quality (Tables S3, S4).

**Table 1:** Characteristics of six included randomized trials.

**Study (year)**	**NCT number**	**Intervention with dosage**	**Patients (number)**	**Comparison**	**Patients (number)**	**Follow up (year)**	**Reported Cognitive impairment**
RE-LY (2009) (Connolly et al., 2009)	NCT00262600	Dabigatran 110 mg twice	5983	Warfarin	5998	2.0	Amnesia; Cognitive disorder; Dementia; Dementia Alzheimer's type; Global amnesia; Memory impairment; Parkinson's disease; Parkinsonism; Vascular dementia
		Dabigatran 150 mg twice	6059				
ROCKET-AF (2011) (Patel et al., 2011)	NCT00403767	Rivaroxaban 20 mg once	7111	Warfarin	7125	1.9	Cognitive disorder; Dementia; Dementia Alzheimer's type; Global amnesia; Parkinson's disease; Parkinsonism; Vascular dementia; Senile Dementia; Sensory Disturbance; Frontotemporal dementia; Altered state of consciousness
ARISTOTLE (2011) (Granger et al., 2011)	NCT00412984	Apixaban 5 mg twice	9088	Warfarin	9052	1.5	Amnesia; Cognitive disorder; Dementia; Dementia Alzheimer's type; Global amnesia; Parkinson's disease; Vascular dementia
AVERROES (2011) (Connolly et al., 2011)	NCT00496769	Apixaban 5 mg twice	2798	Acetylsalicylic acid 81-324 mg	2780	1.1	Dementia; Parkinson's disease
ENGAGE AF-TIMI48 (2013) (Giugliano et al., 2013)	NCT00781391	Edoxaban 60 mg once	7002	Warfarin	7012	2.8	Amnesia; Cognitive disorder; Dementia; Dementia Alzheimer's type; Parkinson's disease; Parkinsonism; Vascular dementia; Senile Dementia; Amnestic disorder; Dementia with Lewy bodies
		Edoxaban 30 mg once	7002				
AXAFA-AFNET 5 (2018) (Kirchhof et al., 2018)	NCT02227550	Apixaban 5 mg twice	318	Vitamin K Antagonist	315	0.25	Cognitive dysfunction

*RE-LY, Randomized Evaluation of Long-term Anticoagulation Therapy; ROCKET AF, Rivaroxaban Once Daily Oral Direct Factor Xa Inhibition Compared with Vitamin K Antagonism for Prevention of Stroke and Embolism Trial in Atrial Fibrillation; ARISTOTLE, Apixaban for Reduction in Stroke and Other Thromboembolic Events in Atrial Fibrillation; AVERROES, A Phase III Study of Apixaban in Patients With Atrial Fibrillation; ENGAGE AF-TIMI 48, Effective Anticoagulation with Factor Xa Next Generation in Atrial Fibrillation–Thrombolysis in Myocardial Infarction 48; AXAFA-AFNET 5, Anticoagulation using the direct factor Xa inhibitor apixaban during Atrial Fibrillation catheter Ablation-Atrial Fibrillation NETwork Association 5*.

**Table 2:** Characteristics of 2 included database studies.

**Study (year)**	**Country**	**Data source**	**Inclusion period**	**Intervention**	**Patients (number)**	**Comparison**	**Patients (number)**	**Adjusted method**	**Adjusted variables**	**Follow up (year)**	**Reported Cognitive impairment (ICD)**
Victoria Jacobs (2016) (Jacobs et al., 2016)	USA	Intermountain Healthcare Clinical Pharmacist Anticoagulation Service	2010.6–2014.12	NOACs	2627	Warfarin	2627	PSM	NR	0.67	Dementia (Alzheimer's, vascular, senile, and non-specified) (290 to 294, 331).
Leif Friberg (2018) (Friberg and Rosenqvist, 2018)	Sweden	Swedish Patient register and the Dispensed Drug register	2006–2014	NOACs	7349	Warfarin	7349	PSM	(1)	3.4	Dementia (F00-03, G051, G300-301, G308-309, G310-312, G318)

*ICD, International Classification of Diseases; NOACs, Non-vitamin K Antagonist Oral Anticoagulants; PSM, propensity score matching; USA, United States of America; NR, not reported; (1): Adjusted variables including Age, gender, number of years since first AF diagnosis, previous ischemic stroke or systemic embolism, intracranial hemorrhage, pulmonary embolism or deep venous thrombosis, myocardial infarction, peripheral artery disease, heart failure, mitral stenosis, mechanical heart valve, other valvular defects, pacemaker or implantable cardioverter/defibrillator (ICD), hypertension, diabetes, renal failure, liver disease, previous hospitalization for major bleeding, Parkinson's disease, hypothyroidism, thyrotoxicosis, chronic obstructive pulmonary disease (COPD), ≥2 hospitalizations for fall accidents, cancer within 3 years, alcohol abuse, baseline use of aspirin, clopidogrel or a non-steroidal anti-inflammatory drug (NSAID), ACE-inhibitor or angiotensin-2 blocker (ARB), statin, beta blocker, class 1 or 3 anti-arrhythmic drugs, digoxin or a diuretic*.

**Table 3:** Patient demographics and clinical characteristics of randomized trials and database studies.

**Study (year)**	**Total number**	**Mean age (year)**	**Male (%)**	**HF (%)**	**Hypertension (%)**	**Diabetes (%)**	**Stroke/TIA/SE (%)**	**Prior MI (%)**	**Prior CHD (%)**	**CHADS_2_/ CHA_2_DS_2_-VASc**
Re-LY (2009) (Connolly et al., 2009)	18040	71.5	63.6	32.0	78.9	23.3	20.0	16.6	NA	2.1
ROCKET-AF (2011) (Patel et al., 2011)	14236	71.2	57.5	62.4	90.5	39.9	54.8	17.3	NA	3.47
ARISTOTLE (2011) (Granger et al., 2011)	18140	69.1	64.7	35.4	NA	25.0	19.4	14.2	30.4	2.1
AVERROES (2011) (Connolly et al., 2011)	5578	69.9	58.5	38.8	86.4	19.6	13.6	NA	NA	2.0
ENGAGE AF-TIMI48 (2013) (Giugliano et al., 2013)	21016	70.6	61.9	57.4	93.6	36.1	28.3	NA	NA	NA
Victoria Jacobs (2016) (Jacobs et al., 2016)	5254	72.4	59	26.6	78.2	30.5	10.8	NA	NA	NA
AXAFA-AFNET 5 (2018) (Kirchhof et al., 2018)	633	64	67	23.7	90.2	12	7.4	NA	NA	2.4
Leif Friberg (2018) (Friberg and Rosenqvist, 2018)	202946	73.7	59.4	31.0	53.2	19.2	21.9	16.5	NA	NA

*HF, heart failure; TIA, transient ischemic attack; SE, systemic embolism; MI, myocardial infarction; CHD, coronary heart disease; NA, not available; RE-LY, Randomized Evaluation of Long-term Anticoagulation Therapy; ROCKET-AF, Rivaroxaban Once Daily Oral Direct Factor Xa Inhibition Compared with Vitamin K Antagonism for Prevention of Stroke and Embolism Trial in Atrial Fibrillation; ARISTOTLE, Apixaban for Reduction in Stroke and Other Thromboembolic Events in Atrial Fibrillation; AVERROES, A Phase III Study of Apixaban in Patients With Atrial Fibrillation; ENGAGE AF-TIMI 48, Effective Anticoagulation with Factor Xa Next Generation in Atrial Fibrillation–Thrombolysis in Myocardial Infarction 48; AXAFA-AFNET 5, Anticoagulation using the direct factor Xa inhibitor apixaban during Atrial Fibrillation catheter Ablation-Atrial Fibrillation NETwork Association 5*.

### Incidence and risk of any and various forms of cognitive impairment

**Figure 1:**
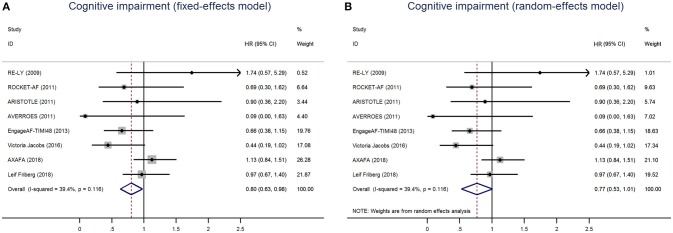
Risk of cognitive impairment of patients receiving NOACs and vitamin K Antagonists (VKAs) or acetylsalicylic acid using fixed-effects model **(A)** or random-effects model **(B)**. HR indicates Hazard ratio; 95%CI indicates 95% confidence interval.

A total of 402 patients (0.41%) were identified with cognitive impairment in the included 6 RCTs, of which 215 (0.39%) were NOACs users and 187 (0.44%) were VKAs or acetylsalicylic acid users. The dominant weight of over 20% came from AXAFA-AFNET 5 trial because it was the first RCT study specially designed to assess the cognitive function, with the incidence of 23.6% in patients with apixaban. After pooling the data of the 2 database studies, the overall results showed a borderline significant association between the use of NOACs and the lower risk of cognitive impairment when compared with VKA/acetylsalicylic acid (HR: 0.80, 95%CI: 0.63–0.98 for fixed-effects model and HR: 0.77; 95%CI: 0.53–1.01 for random-effects model), with no significant heterogeneity across the studies (*I*^2^ = 39.4%, *P* = 0.12) (Figure 1). The results of the narrow definition of cognitive impairment and various forms of cognitive impairment are presented in Figure S2, Table 4. The incidence rate of dementia, Alzheimer's disease, and other individual forms related to AF ranged from 0.01% to 0.07%, and no significant difference was detected between NOACs and comparator regardless of the fixed- or random-effects models.

**Table 4:** Hazard ratios by various forms of cognitive impairment.

**Different forms of cognitive impairment**	**No. of studies**	**With NOACs therapy (%)**	**With comparison therapy (%)**	**HR (95%CI) (fixed model)**	**HR (95%CI) (random model)**	**Homogeneity**	
						***I*^2^ (%)**	***p*-value**
Narrow definition of cognitive impairment^#^	5	32/45043 (0.07%)	27/31967 (0.08%)	0.77 (0.46–1.30)	0.67 (0.39-1.16)	0.0	0.43
Amnesia	3	3/35134 (0.01%)	3/22062 (0.01%)	0.61 (0.17–2.27)	0.679 (0.11–4.20)	19.3	0.29
Cognitive disorder	4	8/35134 (0.02%)	0/22062 (0.00%)	3.90 (0.70–21.77)	3.84 (0.68–21.66)	0.0	0.94
Dementia	5	18/45043 (0.04%)	19/31967 (0.06%)	0.63 (0.34–1.19)	0.60 (0.31–1.15)	0.0	0.68
Dementia Alzheimer's type	4	7/42245 (0.02%)	6/29187 (0.02%)	0.75 (0.25–2.24)	0.83 (0.18–3.83)	18.9	0.3
Global amnesia	3	2/28241 (0.01%)	1/22175 (0.00%)	1.12 (0.21–5.95)	1.143 (0.18–7.25)	0.0	0.63
Memory impairment	1	0/12042 (0.00%)	1/5998 (0.02%)	0.17 (0.01–4.08)	0.17 (0.01–4.08)	–	–
Parkinson's disease	5	8/45043 (0.02%)	12/31967 (0.04%)	0.48 (0.20–1.15)	0.55 (0.22–1.38)	0.0	0.67
Parkinsonism	3	6/33157 (0.02%)	1/20135 (0.00%)	2.14 (0.41–11.1)	1.87 (0.34–10.26)	0.0	0.75
Vascular dementia	4	4/42245 (0.02%)	7/29187 (0.02%)	0.40 (0.13–1.25)	0.39 (0.10–1.42)	0.0	0.47
Senile Dementia	2	2/21115 (0.01%)	0/141373 (0.00%)	2.15 (0.23–19.97)	2.15 (0.22–20.43)	0.0	0.76
Sensory Disturbance	1	1/7111 (0.01%)	0/7125 (0.00%)	3.01 (0.12–73.78)	3.01 (0.12–73.78)	–	–
Frontotemporal dementia	1	1/7111 (0.01%)	0/7125 (0.00%)	3.01 (0.12–73.78)	3.01 (0.12–73.78)	–	–
Altered state of consciousness	1	0/7111 (0.00%)	1/7125 (0.01%)	0.33 (0.01–8.20)	0.33 (0.01–8.20)	–	–
Amnestic disorder	1	1/14004 (0.01%)	0/7012 (0.00%)	1.50 (0.06–36.87)	1.5 (0.06–36.87)	–	–
Dementia with Lewy bodies	1	0/14004 (0.00%)	2/7012 (0.03%)	0.10 (0.01–2.09)	0.1 (0.01–2.09)	–	–

*NOACs, Non-vitamin K Antagonist Oral Anticoagulants; HR, Hazard Ratio; 95%CI: 95% confidence interval. ^#^Narrow definition of cognitive impairment including dementia, dementia Alzheimer's type, vascular dementia, senile dementia, frontotemporal dementia, and dementia with Lewy bodies*.

### Subgroups, sensitivity, and meta-regression analyses

Table 5 summarized an overall result of the subgroup analysis, and the detailed results by study type, individual NOACs, and the duration of follow-up are shown in Figures S3–S5. Regarding the duration of follow-up, the results of over 1 year showed that the patients with NOACs were associated with a borderline lower risk of cognitive impairment in comparison to patients with VKAs/acetylsalicylic acid (HR: 0.76, 95%CI: 0.54–0.99 for fixed-effects model and HR: 0.76, 95%CI: 0.54–0.99 for random-effects model), and no significant interaction was detected between the subgroups (*P*_interaction_ = 0.60). The results of the study types and individual NOACs subgroup failed to show a significantly low risk of cognitive impairment (*P*_interaction_ = 0.55 for study type and *P*_interaction_ = 0.51 for individual NOACs). The sensitivity analyses also failed to identify any individual trial as having influenced the results to a significant extent (Table 6, Figures S6–S9). Furthermore, the meta-regression analysis failed to detect any potential confounding of clinical characteristics on the risk of cognitive impairment (Table S5).

**Table 5:** Subgroup analyses.

**Subgroup**	**No. of studies**	**With NOACs therapy**	**With comparison therapy**	**HR (95%CI) (fixed model)**	**HR 95%CI) (random model)**	**Homogeneity**		***P* for interaction**
						***I*^2^ (%)**	**p value**	
**Study type**								0.55
RCTs	6	136/45361(0.30%)	120/32282(0.37%)	0.85 (0.63–1.06)	0.79 (0.48–1.11)	35.1	0.17	
Database studies	2	79/9976 (0.79%)	67/9976(0.67%)	0.74 (0.47–1.01)	0.72 (0.20–1.23)	71.4	0.06	
**NOACs**								0.51
Dabigatran	1	14/12042(0.11%)	4/5998(0.07%)	1.74 (0.57–5.29)	1.74 (0.57–5.29)	–	–	
Rivaroxaban	1	9/7111(0.13%)	13/7125(0.18%)	0.69 (0.30–1.62)	0.69 (0.30–1.62)	–	–	
Apixaban	3	84/12204(0.69%)	81/12147(0.67%)	0.97 (0.68–1.26)	0.77 (0.14–1.41)	62.7	0.07	
Edoxaban	1	29/14004(0.21%)	22/7012(0.31%)	0.66 (0.38–1.15)	0.66 (0.38–1.15)	–	–	
**Follow-up**								0.60
>1 year	6	132/52392(0.25%)	103/39316(0.26%)	0.76 (0.54–0.99)	0.76 (0.54–0.99)	0.00	0.43	
<1 year	2	83/2945(2.82%)	84/2942(2.9%)	0.86 (0.60–1.12)	0.80 (0.13–1.46)	84.2	0.01	

*RCTs, randomized controlled trials; NOACs, Non-vitamin K Antagonist Oral Anticoagulants; HR, Hazard Ratio; 95%CI: 95% confidence interval*.

**Table 6:** Sensitive analyses.

**Study omitted**	**HR (95%CI) (fixed model)**	**HR (95%CI) (random model)**
RE-LY (2009) (Connolly et al., 2009)	0.80 (0.63–0.97)	0.76 (0.51–1.01)
ROCKET-AF (2011) (Patel et al., 2011)	0.81 (0.64–0.99)	0.77 (0.50–1.04)
ARISTOTLE (2011) (Granger et al., 2011)	0.80 (0.63–0.98)	0.76 (0.49–1.02)
AVERROES (2011) (Connolly et al., 2011)	0.84 (0.66–1.01)	0.82 (0.60–1.05)
EngageAF-TIMI48 (2013) (Giugliano et al., 2013)	0.84 (0.65–1.03)	0.79 (0.50–1.08)
Victoria Jacobs (2016) (Jacobs et al., 2016)	0.88 (0.69–1.07)	0.85 (0.61–1.09)
AXAFA-AFNET 5 (2018) (Kirchhof et al., 2018)	0.69 (0.49–0.89)	0.68 (0.47–0.90)
Leif Friberg (2018) (Friberg and Rosenqvist, 2018)	0.76 (0.57–0.95)	0.72 (0.43–1.00)
**STUDY EXCLUDED**		
Catheter ablation studies	0.69 (0.49–0.89)	0.68 (0.47–0.90)
Acetylsalicylic acid as control studies	0.84 (0.66–1.01)	0.82 (0.60–1.05)
Low dosage arms of NOACs	0.84 (0.66–1.02)	0.80 (0.55–1.05)
**STUDY ADDED**		
Adding MRI sub-study in AXAFA-AFNET 5 trial	0.85 (0.69–1.01)	0.83 (0.61–1.04)

*HR, Hazard Ratio; 95%CI: 95% confidence interval; RE-LY, Randomized Evaluation of Long-term Anticoagulation Therapy; ROCKET AF, Rivaroxaban Once Daily Oral Direct Factor Xa Inhibition Compared with Vitamin K Antagonism for Prevention of Stroke and Embolism Trial in Atrial Fibrillation; ARISTOTLE, Apixaban for Reduction in Stroke and Other Thromboembolic Events in Atrial Fibrillation; AVERROES, A Phase III Study of Apixaban in Patients With Atrial Fibrillation; ENGAGE AF-TIMI 48, Effective Anticoagulation with Factor Xa Next Generation in Atrial Fibrillation–Thrombolysis in Myocardial Infarction 48; AXAFA-AFNET 5, Anticoagulation using the direct factor Xa inhibitor apixaban during Atrial Fibrillation catheter Ablation-Atrial Fibrillation NETwork Association 5*.

### Publication bias

A visual inspection of the funnel plot showed a relative symmetry despite the presence of a small sample effect (AVERROES trial), suggesting that the publication bias was not a concern overall (Figure S10).

## Discussion

Effective oral anticoagulation reduces the burden of embolic stroke in patients with AF and may bring about correspondingly preserved effects of cognitive function. NOACs, due to favorable property of thromboembolism prophylaxis and reduced bleeding risk, have been recommended as an optimal alternative to warfarin. It is plausible to assume that the NOACs might lead to a better protection against AF-related cognitive dysfunction than warfarin. In support of this hypothesis, we have performed the first systematic review to pool current evidences from RCTs and high-quality observational database studies for evaluating the association between the use of NOACs and the risk of cognitive impairment. The results indicated that the use of NOACs might have a potential tendency to decrease the risk of cognitive impairment compared with VKAs or acetylsalicylic acid.

Although evidence suggests that AF is associated with a high risk of cognitive dysfunction and dementia, the precise mechanism is not fully known. The relationship between AF and cognitive disorder might occur through a variety of pathological mechanisms. One of the leading potential mechanisms is the occurrence of stroke in AF patients, either overt or silent stroke (Dagres et al., 2018). Stroke is recognized as the most feared complication of AF, and could result from static blood produced by fibrillation of atrium, ultimately leading to thrombus formation and embolism to the brain (de Bruijn et al., 2015; Kamel et al., 2016). It is of interest to know that silent cerebral infarction occurs more frequently than clinical stroke and is more common in AF patients, and it affects the frontal circuit components that are essential for the executive function (Konno et al., 1997). Meanwhile, a recent *post-hoc* analysis of 31,546 AF patients observed that cognitive impairment was related to AF during long-term follow-up even in the absence of clinical stroke (Marzona et al., 2012). Moreover, a meta-analysis of 8 observational studies found that AF was independently associated with an increased risk of dementia (HR:1.42; 95% CI: 1.17–1.72, *P* < 0.001) in patients without acute stroke. Therefore, another speculation was raised that vascular dementia, which was similar to the pattern of silent infarct distribution, might be an obvious contributor to cognitive impairment, encompassing both multi-infarct dementia and small vessel disease dementia (Ott et al., 1997; Thacker et al., 2013). Beyond that, a lower cardiac output in AF could lead to chronic cerebral hypoperfusion, which may in turn cause damage to the brain (de Bruijn et al., 2015). Furthermore, AF was also identified to be a risk factor for Alzheimer's disease, the most common type of dementia (Bunch et al., 2010; Poggesi et al., 2015).

Regarding the probable mechanisms described above, stroke prevention, particularly effective oral anticoagulation, might lead to preserved cognitive function. One retrospective registry study including 44,106 AF patients suggested that the patients on anticoagulant treatment (94% using warfarin) were associated with 29% lower risk of dementia than those without anticoagulant treatment (HR: 0.71, 95% CI: 0.68–0.74) (Friberg and Rosenqvist, 2018). Nevertheless, TTR of warfarin might also be a factor inferencing the risk of dementia. Studies showed that the percent of time exposed to over-anticoagulation could increase the dementia risk in AF patients receiving warfarin (Jacobs et al., 2015). High TTR value of warfarin, meaning effective anticoagulation, requires daily compliance, periodic adjustment of dose, and knowledge of drug and food interactions (Flaker et al., 2010). Therefore, it is difficult to be understood in elderly AF patients and patients with cognitive dysfunction. Over the last decade, NOACs have been developed and revealed to be non-inferior to VKAs for stroke prevention, with more stable anticoagulant effects in the long-term treatment. Moreover, NOACs are related to lower risk of intracranial micro-hemorrhage compared with warfarin (Ruff et al., 2014; Jacobs et al., 2016). Regretfully, the question of whether NOACs could play a protective role on cognitive function in AF patients remains unanswered. AXAFA-AFNET 5 trial was published in March 2018, which was the first RCT to evaluate the cognitive function with apixaban vs. VKA in patients undergoing AF ablation (Kirchhof et al., 2018). Montreal Cognitive Assessment Test (MoCA) increased by a median of +1.0 unit at the end of the follow-up but without differences between apixaban and VKA (Kirchhof et al., 2018). Similarly, among 323 analyzable brain magnetic resonance imagines (MRIs), acute brain MRI lesions were observed in 27.2% patients allocated to apixaban and 24.8% patients allocated to VKA, with consistent distribution between the two groups (*P* = 0.635) (Kirchhof et al., 2018).

It is noteworthy that high-quality observational studies could provide more valuable evidence on additional risk of novel agents, especially when there are gaps in evidence from RCTs. A database study currently provided some optimism that AF patients taking NOACs were associated with a lower risk of cerebral ischemic events and new-onset dementia than those taking warfarin (Jacobs et al., 2016). For a strong argument on this issue, we collected over 90,000 patients by integrating 6 RCTs and 2 high-quality real-world studies to estimate the risk of cognitive impairment with NOACs. The included studies were of modest to high quality, and the methodology of the present study was made rigorous by using the fixed- and random-effects models simultaneously. The sensitivity analysis confirmed the robustness of the results, and no potentially clinical confounding was identified by meta-regression analysis. The primacy results clearly revealed a borderline significant result in favor of NOACs, whereas the result of narrow definition of cognitive impairment failed to show a significant result. For the above two analyses, the definition was different, with primacy results involving all forms of cognitive disorder reported in the studies, and the narrow definition considering more accurate cognitive dysfunction with various types of dementia. The inconsistent results might come from the low incidence of AF-related cognitive impairment and the insufficient patient samples, meaning that further RCTs and database studies were needed to strengthen the association of lower risk of cognitive impairment with NOACs vs. VKAs. Interestingly, the protective effects of NOACs on cognitive function were detected in patients requiring long-term anticoagulation treatment (>1 year), which might imply a sustaining protection against ischemic stroke during the process of NOACs treatment.

Several limitations should be addressed in our study. Firstly, the inevitable heterogeneity between RCTs and observational studies need to be considered even though there is demonstration of non-significant heterogeneity across studies. Different confounding factors were adjusted in observational studies, which made it challenging to compare the results across the studies. Nevertheless, we have performed a meta-regression analysis to estimate the potential effect modifiers in clinical characteristics, and the results failed to identify any significant confounding on outcomes. Secondly, no included RCTs were especially designed to assess the cognitive function of NOACs except for the AXAFA-ASNET 5 study, thus the absence of a clear and uniform definition of cognitive impairment and incomprehensive collection of cognitive data across trials might introduce certain bias. Meanwhile, the mean duration of follow-up was relatively insufficient, which may underestimate the protective effects of NOACs on cognitive function. Thirdly, 20 RCTs and 50 database studies were excluded from the analysis due to the unavailable data of cognitive function, which might reduce the power of statistics.

## Conclusions

The results indicated a lower tendency of cognitive impairment risk on NOACs when compared with VKA or acetylsalicylic acid in patients with AF. Further designs of RCTs on evaluation of cognitive function and long-term observational studies based on real-world experience are necessary for obtaining a robust association between NOACs and cognitive impairment risk.

## Author contributions

X-YL, H-WL, and JP are the guarantors of the entire manuscript. CZ and Z-CG contributed to the study conception and design, data acquisition, analysis, and interpretation, drafting of the manuscript, critical revision of the manuscript for important intellectual content, and final approval of the version to be published. LS, M-MP, and Y-DY contributed to the data acquisition, analysis, and interpretation.

### Conflict of interest statement

The authors declare that the research was conducted in the absence of any commercial or financial relationships that could be construed as a potential conflict of interest.
