# Supplementary material for: Non-vitamin K Antagonist Oral Anticoagulants and Cognitive Impairment in Atrial Fibrillation: Insights From the Meta-Analysis of Over 90,000 Patients of Randomized Controlled Trials and Real-World Studies
Source: Front Aging Neurosci. 2018 Oct 2;10:258. doi: 10.3389/fnagi.2018.00258 (PMC6176145; doi:10.3389/fnagi.2018.00258)
Supplement: Supplementary file 1 [file Data_Sheet_1.DOCX]

Supplementary Material

**Title:** Non-vitamin K Antagonist Oral Anticoagulants and Cognitive Impairment in Atrial Fibrillation: Insights from the Meta-analysis of Over 90,000 Patients of Randomized Controlled Trials and Real-World Studies

**Running Title**: Cognitive Impairment of NOACs in AF

**Authors:** Chi Zhang, MD^1#^; Zhi-Chun Gu, MD^1#^; Long Shen, MD^2^; Mang-Mang Pan, MD^1^; Yi-Dan Yan, MD^1^; Jun Pu, MD, PhD, FESC, FACC^2^; Xiao-Yan Liu, MD^1*^; Hou-Wen Lin, MD, PhD^1*^

***Correspondence Author:**

Xiao-Yan Liu, MD; E-mail: liuxiaoyanrenji@163.com

OR

Hou-Wen Lin, MD, PhD; E-mail: linhouwenrenji@163.com

**Index**

**Tables**

Table S1. Reasons for excluded randomized controlled trials

Table S2. Reasons for excluded database studies

Table S3. Quality assessment of RCTs

Table S4. Quality assessment of database studies

Table S5. Meta-regression analysis

**Figures**

FigureS1. Flow diagram for the selection of eligible Studies

Figure S2. Narrow definition of cognitive impairment

Figure S3. Subgroup analysis according to individual NOACs

Figure S4. Subgroup analysis according to follow-up

Figure S5. Subgroup analysis according to study type

Figure S6. Sensitivity analysis by excluding catheter ablation study

Figure S7. Sensitivity analysis by excluding study involved acetylsalicylic acid as control

Figure S8. Sensitivity analysis by excluding low dosage arms of NOACs

Figure S9. Sensitivity analysis by adding the data of magnetic resonance imaging (MRI) sub-study in AXAFA trial

Figure S10. Funnel plot

**Table S1. Randomized controlled trials excluded for a) one arm study and b) not reporting cognitive impairment data.**

| **NCT number** | **Phase** | **Study name** | **Drugs** | **Condition** | **Reason for exclusion** |
| --- | --- | --- | --- | --- | --- |
| NCT01546883 | 4 | DEPAF | Dabigatran | AF | a |
| NCT02348723 | 4 | RE-CIRCUIT | Dabigatran | AF | b |
| NCT00808067 | 3 | RELY-ABLE | Dabigatran | AF | a |
| NCT01896297 | 4 | NA | Dabigatran | AF and CKD | a |
| NCT01493557 | 4 | NA | Dabigatran | AF | b |
| NCT01868243 | 2, 3 | DAWA | Dabigatran | AF with bioprosthetic replacement | b |
| NCT01227629 | 2 | PETRO | Dabigatran | AF | b |
| NCT01136408 | 2 | AF-DABIG-VKA-JAPAN | Dabigatran | AF | b |
| NCT01674647 | 3 | X-VERT | Rivaroxaban | AF and cardioversion | b |
| NCT01729871 | 3 | VENTURE-AF | Rivaroxaban | AF with ablation | b |
| NCT00494871 | 3 | J-ROCKET-AF | Rivaroxaban | AF | b |
| NCT00412984 | 2 | ARISTOTLE-J | Apixaban | AF | b |
| NCT01706146 | 4 | REACT COM | Apixaban | AF | a |
| NCT01857622 | 3 | NA | Edoxaban | AF and CKD | a |
| NCT00829933 | 2 | AF-EDOX-VKA-JAPAN | Edoxaban | AF | b |
| NCT00806624 | 2 | AF-EDOX-VKA-ASIA | Edoxaban | AF | b |
| NCT00504556 | 2 | AF-EDOX-VKA-MULTI | Edoxaban | AF | b |
| NCT00742859 | 2 | EXPLORE-XA | Betrixaban | AF | b |
| [NCT01830543](http://clinicaltrials.gov/show/NCT01830543) | 4 | PIONEER AF-PCI | Rivaroxaban | AF and PCI | b |
| NCT02164864 | 4 | RE-DUAL PCI | Dabigatran | AF and PCI | b |

AF: atrial fibrillation; CKD: chronic kidney disease; NA: not available; PCI: percutaneous coronary intervention; a: one arm study; b: not reporting cognitive impairment

**Table S2. Database studies excluded for no cognitive impairment data reported**

| **Study (year)** | **Indication** | **Country** | **Data Source** | **Inclusion Period** |
| --- | --- | --- | --- | --- |
| Larsen (2013)([Larsen et al., 2013](#_ENREF_33)) | AF | Denmark | The Danish Civil Registration system; the National Patient Register; the Danish National Prescription Registry | 2011.8.1-2012.12.31 |
| Sørensen (2013)([Sorensen et al., 2013](#_ENREF_49)) | AF | Denmark | The Danish Civil Registration system; the National Patient Register; the Danish National Prescription Registry | 1995.1.1-2011.12..31 |
| Alonso (2014)([Alonso et al., 2014](#_ENREF_2)) | AF | U.S.A. | The Truven Health MarketScan Commercial Claims and Encounters Database and the Medicare Supplemental and Coordination of Benefits Database | 2009.1.1-2012.12.31 |
| Larsen (2014)([Larsen et al., 2014a](#_ENREF_31)) | AF | Denmark | The Truven Health MarketScan Commercial Claims and Encounters Database and the Medicare Supplemental and Coordination of Benefits Database | 2009.8.1-2013.3.30 |
| Larsen (2014)([Larsen et al., 2014b](#_ENREF_32)) | AF | Denmark | The Truven Health MarketScan Commercial Claims and Encounters Database and the Medicare Supplemental and Coordination of Benefits Database | 2009.8.1-2013.3.30 |
| Vaughan Sarrazin (2014)([Vaughan Sarrazin et al., 2014](#_ENREF_54)) | AF | U.S.A. | National Veterans Affairs administrative encounter and pharmacy data | 2010-2012 |
| Abraham (2015)([Abraham et al., 2015](#_ENREF_1)) | AF | U.S.A. | Optum Labs Data Warehouse | 2010.12.1-2013.9.30 |
| Chang (2015)([Chang et al., 2015](#_ENREF_6)) | AF | U.S.A. | IMS Health Life Link Health Plan Claims Database | 2010.10.1-2012.3.31 |
| Hernandez (2015)([Hernandez et al., 2015](#_ENREF_20)) | AF | Spain | The Centers for Medicare and Medicaid Services | 2010.10.1-2011.10.31 |
| Ho (2015)([Ho et al., 2015](#_ENREF_24)) | AF | Hong Kong | Queen Mary Hospital (8754 patients) | 1997.7-2011.12 |
| Lauffenburger (2015)([Lauffenburger et al., 2015](#_ENREF_35)) | AF | U.S.A. | Truven Health MarketScan Commercial Claims and Encounters and Medicare Supplement databases | 2010.10-2012.12 |
| Maura (2015)([Maura et al., 2015](#_ENREF_41)) | AF | France | The French National Insurance information system; the French Hospital Discharge database | NA |
| Staerk (2015)([Staerk et al., 2015](#_ENREF_52)) | AF | Denmark | Danish Civil Registration system; the National Patient Register; the Danish National Prescription Registry | 2011.8.22-2012.12.31 |
| Avgil-Tsadok (2016)([Avgil-Tsadok et al., 2016](#_ENREF_3)) | AF | Canada | Provincial hospital discharge database, physician and prescription claims database | 1999.1.1-2013.3.31 |
| Chan (2016)([Chan et al., 2016](#_ENREF_5)) | AF | Taiwan | Taiwan National Health Insurance Research Database (NHIRD) | 1996.1.1-2013.12.31 |
| Ellis (2016)([Ellis et al., 2016](#_ENREF_11)) | AF | Israel | The computerized database of the Israeli Clalit Health Services Healthcare Organization | 2011.1.1-2013.12.1 |
| Gorst-Rasmussen (2016)([Gorst-Rasmussen et al., 2016](#_ENREF_16)) | AF | Denmark | Danish Civil Registration system; the National Patient Register; the Danish National Prescription Registry | 2012.2-2014.8 |
| Harel (2016)([Harel et al., 2016](#_ENREF_19)) | AF | Canada | The Ontario Public Drug Benefit Program Database, Canadian Institute for Health Information Discharge Abstract Database, National Ambulatory Care Reporting System | NA |
| Larsen (2016)([Larsen et al., 2016](#_ENREF_34)) | AF | Denmark | Danish Civil Registration system; the National Patient Register; the Danish National Prescription Registry | 2011.8.1-2015.12.30 |
| Lip (2016)([Lip et al., 2016b](#_ENREF_39)) | AF | U.S.A. | US Truven MarketScan data | 2013.1.1-2013.12.31 |
| Lip (2016)([Lip et al., 2016a](#_ENREF_38)) | AF | U.S.A. | US Truven MarketScan data | 2012.1.1-2014.12.31 |
| Nishtala (2016)([Nishtala et al., 2016](#_ENREF_44)) | AF | New Zealand | The National Minimum Dataset (NMDS) | 2011.7.1-2012.12.31 |
| Yao (2016)([Yao et al., 2016](#_ENREF_56)) | AF | U.S.A. | Optum Labs Data Warehouse | 2010.10.1-2015.6.30 |
| Yap (2016)([Yap et al., 2016](#_ENREF_57)) | AF | Malaysia | Single-center retrospective cohort registry | 2009.1-2013.12 |
| Yavuz (2016)([Yavuz et al., 2016](#_ENREF_58)) | AF | Turkey | Consecutive NVAF patients in 4 centers | 2013.3-2014.11 |
| Korenstra (2016)([Korenstra et al., 2016](#_ENREF_29)) | AF | Netherlands | Martini Hospital Groningen | 2010.1.1-2012.12.31 |
| Cha (2017)([Cha et al., 2017](#_ENREF_4)) | AF | Korea | The Korean National Health Insurance Service database | 2014.1-2015.12 |
| Coleman (2017)([Coleman et al., 2017](#_ENREF_8)) | AF | U.S.A. | US Truven MarketScan data | 2012.1-2015.6 |
| Friberg (2017)([Friberg and Oldgren, 2017](#_ENREF_12)) | AF | Sweden | The National Swedish Patient Register, Dispensed Drug Register, Cause of Death Register and the socioeconomic longitudinal integration database for health insurance and labor market studies (LISA) register | 2011.12.1-2014.12.31 |
| Gieling (2017)([Gieling et al., 2017](#_ENREF_14)) | AF | British | The Clinical Practice Research Datalink (CPRD) | 2008.3.18-2014.10.1 |
| Halvorsen (2017)([Halvorsen et al., 2017](#_ENREF_18)) | AF | Norway | The Norwegian Patient Registry and the Norwegian Prescription Database | 2013.1.1-2015.6.30 |
| Hernandez (2017)([Hernandez and Zhang, 2017](#_ENREF_21)) | AF | U.S.A. | The Centers for Medicare and Medicaid Services | 2010-2013 |
| Hernandez (2017)([Hernandez et al., 2017a](#_ENREF_22)) | AF | U.S.A. | The Centers for Medicare and Medicaid Services | 2010.10.19-2012.12.31 |
| Hernandez (2017)([Hernandez et al., 2017b](#_ENREF_23)) | AF | U.S.A. | The Centers for Medicare and Medicaid Services | 2013.1.1-2014.12.31 |
| Hohnloser (2017)([Hohnloser et al., 2017](#_ENREF_25)) | AF | Germany | German claims database | 2013.1.1-2015.3.31 |
| Kohsaka (2017)([Kohsaka et al., 2017](#_ENREF_28)) | AF | Japan | De-identified data from health claims and Diagnosis Procedure Combination | 2011.3.1-2016.3.31 |
| Lamberts (2017)([Lamberts et al., 2017](#_ENREF_30)) | AF | Denmark | Danish Civil Registration system; the National Patient Register; the Danish National Prescription Registry | 2011.8.22-2015.12.31 |
| Li (2017)([Li et al., 2017](#_ENREF_36)) | AF | Hong Kong | Hospital-based AF registry | 2008.1-2014.12 |
| Lin (2017)([Lin et al., 2017](#_ENREF_37)) | AF | U.S.A. | IMS Parametric Plus database | 2013.1.1-2015.9.30 |
| Lip (2017)([Lip et al., 2017](#_ENREF_40)) | AF | Denmark | Danish Civil Registration system; the National Patient Register; the Danish National Prescription Registry | NA |
| Nielsen (2017)([Nielsen et al., 2017](#_ENREF_43)) | AF | Denmark | Danish Civil Registration system; the National Patient Register; the Danish National Prescription Registry | 2011.8-2016.2 |
| Palamaner Subash Shantha (2017)([Palamaner Subash Shantha et al., 2017](#_ENREF_46)) | AF | U.S.A. | The Centers for Medicare and Medicaid Services patient records and linked data sources | 2011.11.1-2013.10.31 |
| Song (2017)([Song et al., 2017](#_ENREF_48)) | AF | U.S.A. | MarketScan Commercial Claims and Encounters and Medicare Supplemental and Coordination of Benefits Databases | 2010.1.1-2013.12.31 |
| Staerk (2017)([Staerk et al., 2017a](#_ENREF_50)) | AF | Denmark | Danish Civil Registration system; the National Patient Register; the Danish National Prescription Registry | 2011.8.22-2015.12.31 |
| Staerk (2017)([Staerk et al., 2017b](#_ENREF_51)) | AF | Denmark | Danish Civil Registration system; the National Patient Register; the Danish National Prescription Registry | 2012.3.1-2016.12.31 |
| Stolk (2017)([Stolk et al., 2017](#_ENREF_53)) | AF | British | The Clinical Practice Research Datalink (CPRD) | 2008.3.18-2014.6.30 |
| Yamashita (2017)([Yamashita et al., 2017](#_ENREF_55)) | AF | Japan | The Fushimi AF Registry | 2011.3-2015.11 |
| Naganuma (2017)([Naganuma et al., 2017](#_ENREF_42)) | AF | Japan | NA | 2011.3-2013.12 |
| Coleman (2016)([Coleman et al., 2016](#_ENREF_7)) | AF | U.S.A. | US Truven MarketScan data | 2012.1-2014.10 |
| Norby (2017)([Norby et al., 2017](#_ENREF_45)) | AF | U.S.A. | Truven Health MarketScan Commercial Claims and Encounters Database | 2010.1.1-2014.12.31 |

**Table S3. Quality assessment of RCTs**

| **Study** | **Random sequence generation** | **Allocation concealment** | **Blinding of participants and personnel** | **Blinding of outcome assessment** | **Incomplete outcome data** | **Selective reporting** | **Other bias** |
| --- | --- | --- | --- | --- | --- | --- | --- |
| RE-LY (2009)([Connolly et al., 2009](#_ENREF_10)) | Low | Low | High | Low | Low | Low | Low |
| ROCKET-AF (2011)([Patel et al., 2011](#_ENREF_47)) | Low | Low | Low | Low | Low | Low | Low |
| ARISTOTLE (2011)([Granger et al., 2011](#_ENREF_17)) | Low | Low | Low | Low | Low | Low | Low |
| AVERROES (2011)([Connolly et al., 2011](#_ENREF_9)) | Low | Low | Low | Low | Low | Low | Low |
| EngageAF-TIMI48 (2013)([Giugliano et al., 2013](#_ENREF_15)) | Low | Low | Low | Low | Low | Low | Low |
| AXAFA-AFNET 5 (2018)([Kirchhof et al., 2018](#_ENREF_27)) | Low | Low | High | Low | Low | Low | Low |

Low: low risk; unclear: unclear risk; High: high risk

**Table S4. Quality assessment of database studies**

| **Study** | **Selection bias** | **Bias due to residual confounding** | **Bias due to time-varying covariates/information censoring** | **Bias due to selective reporting of study outcomes** |
| --- | --- | --- | --- | --- |
| Victoria Jacobs (2016)([Jacobs et al., 2016](#_ENREF_26)) | Low | Moderate | Moderate | Low |
| Leif Friberg (2018)([Friberg and Rosenqvist, 2018](#_ENREF_13)) | Low | Low | Low | Low |

Low: low risk; Moderate: moderate risk: unclear risk; High: high risk

**Table S5. Meta-regression analysis**

| **Variable** | **P value** |
| --- | --- |
| Mean age (year) | 0.61 |
| Male (%) | 0.58 |
| Heart failure | 0.54 |
| Hypertension | 0.93 |
| Diabetes | 0.42 |
| Stroke/ transient ischemic attack/SE | 0.56 |
| Prior myocardial infarction | 0.84 |
| CHADS2/ CHA2DS2-VAS | 0.92 |
| Follow-up | 0.84 |

P values: it is the results of meta-regression for the relationship between each variable and the outcome

**
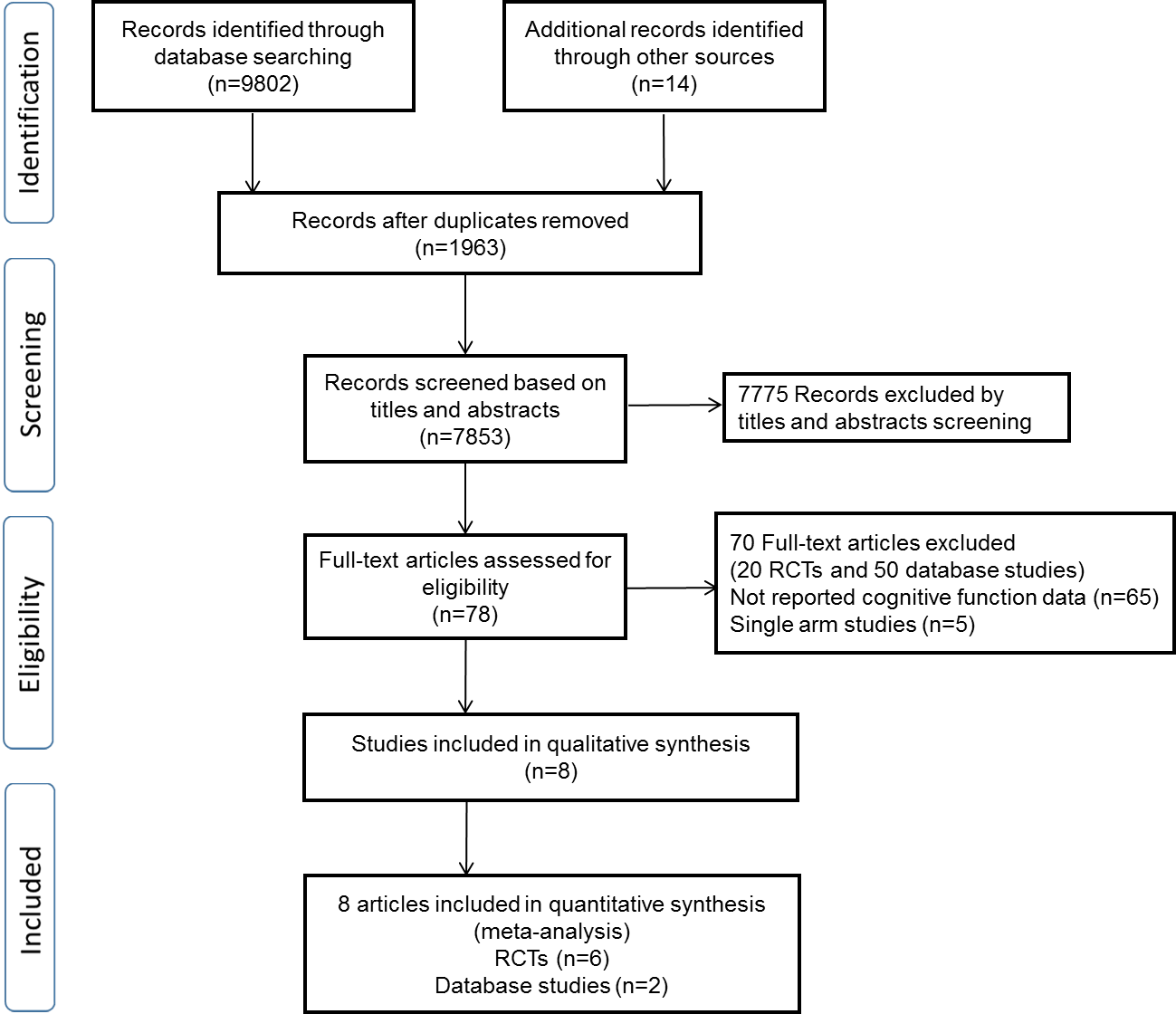
**

**FigureS1. Flow diagram for the selection of eligible Studies.**

**
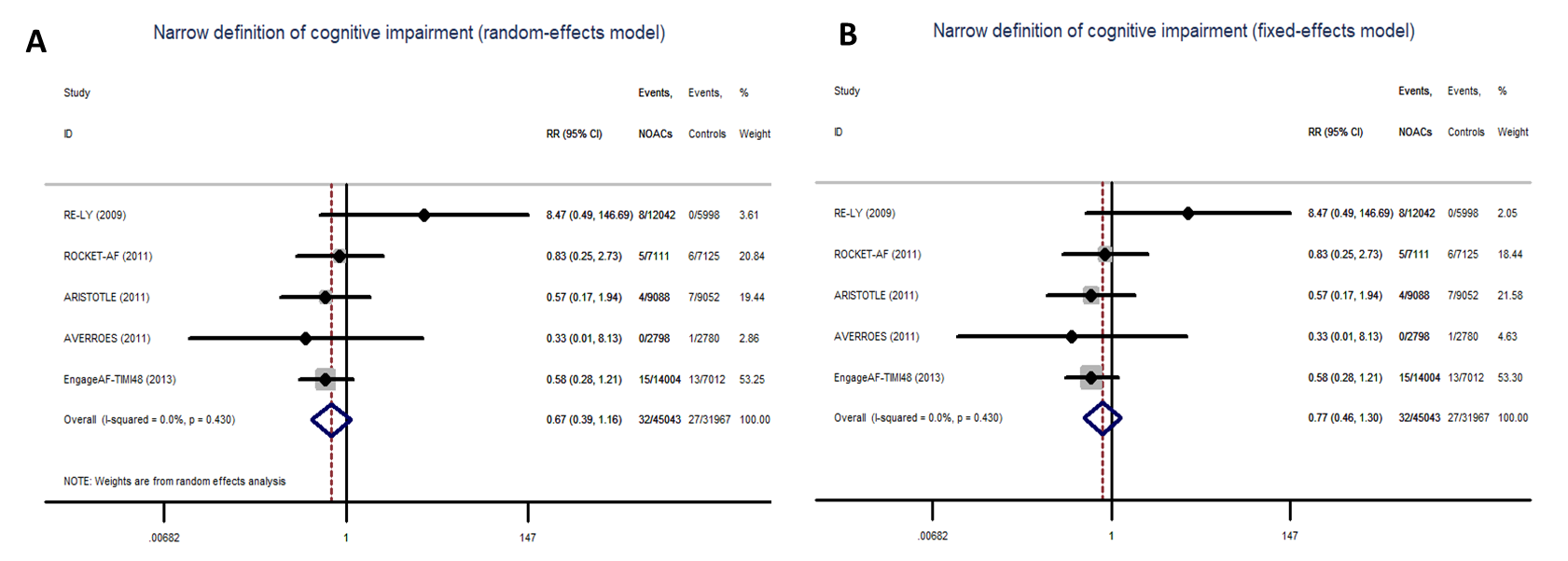
**

**Figure S2.** Narrow definition of cognitive impairment using fixed-effect model (A) and random-effect model (B)


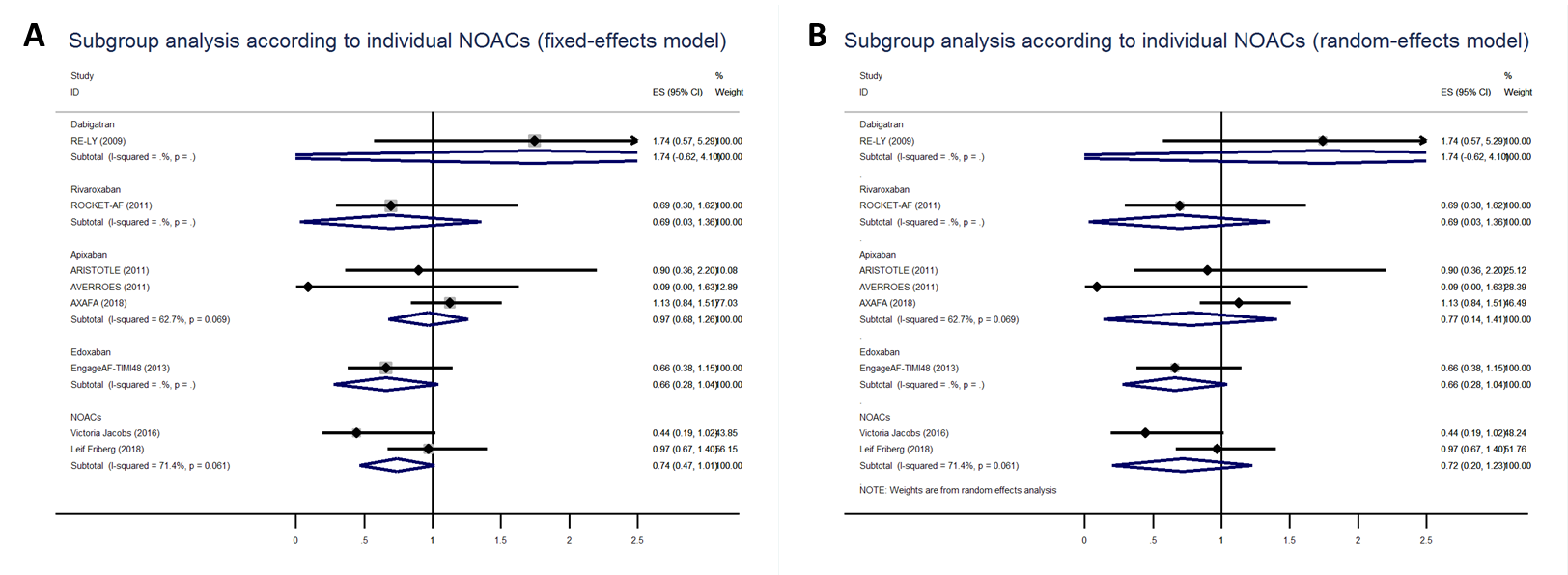
**Figure S3.** Subgroup analysis according to individual NOACs using fixed-effect model (A) and random-effect model (B).


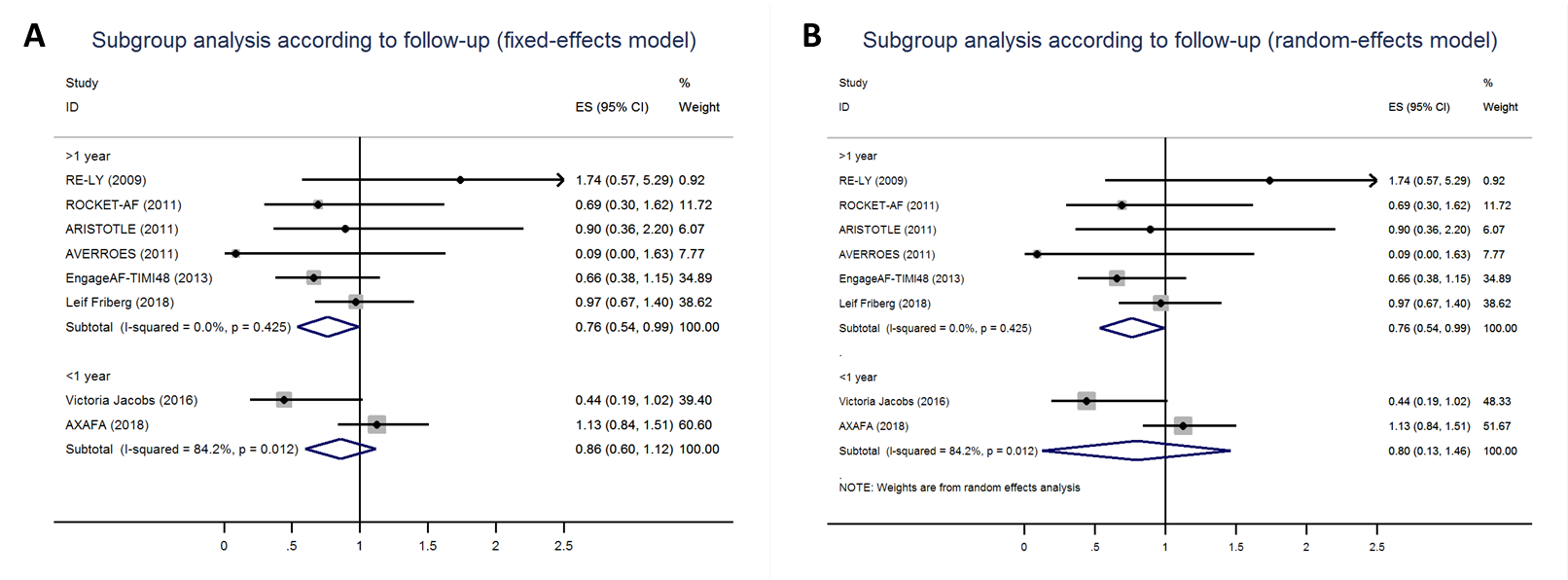
**Figure S4.** Subgroup analysis according to follow-up using fixed-effect model (A) and random-effect model (B).


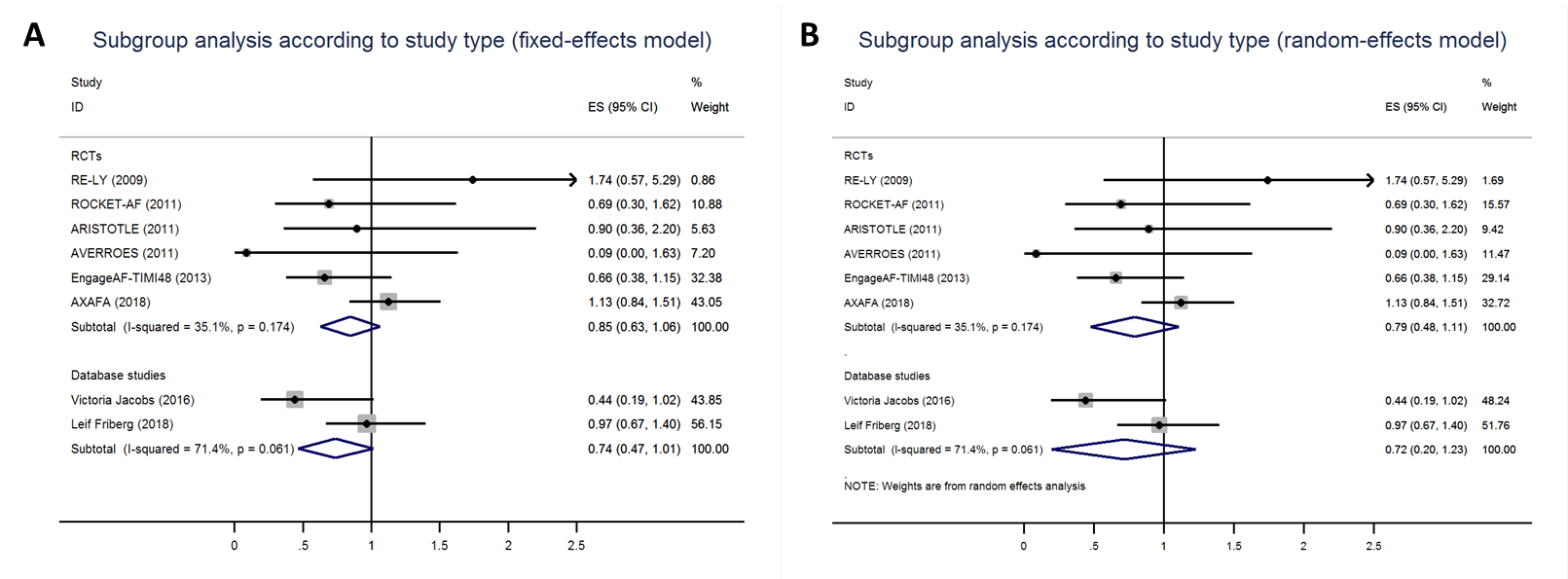
**Figure S5.** Subgroup analysis according to study type using fixed-effect model (A) and random-effect model (B).


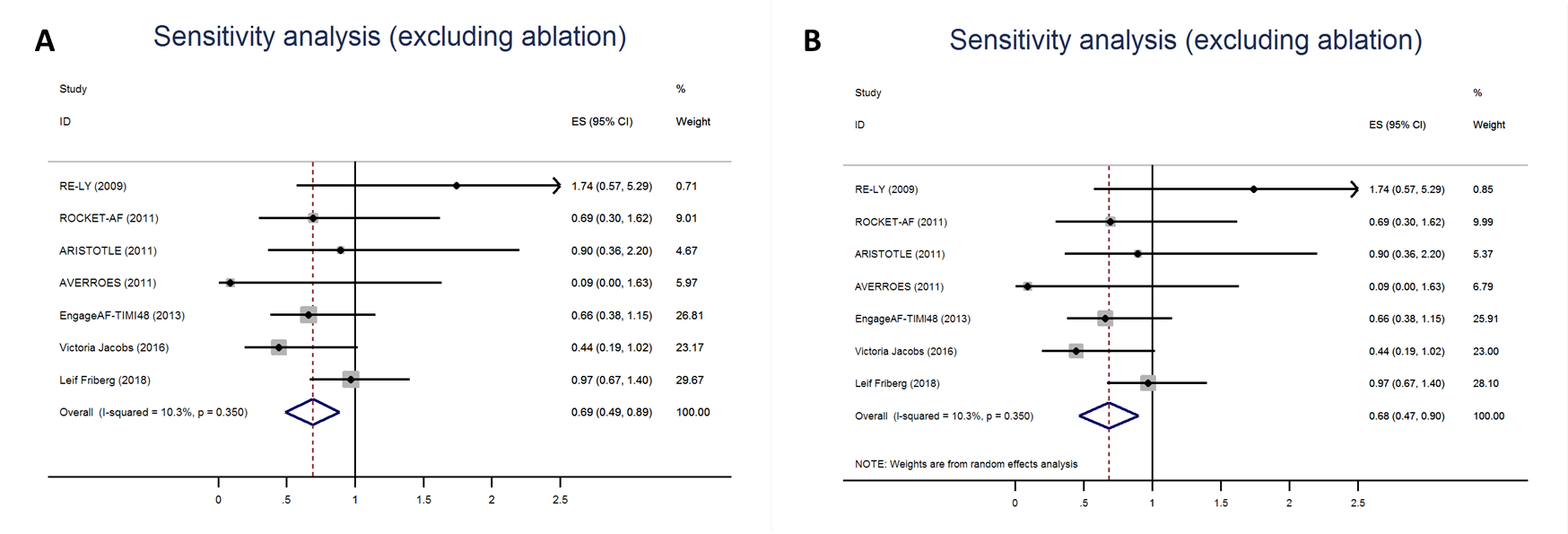
**Figure S6.** Sensitivity analysis by excluding catheter ablation study using fixed-effect model (A) and random-effect model (B).


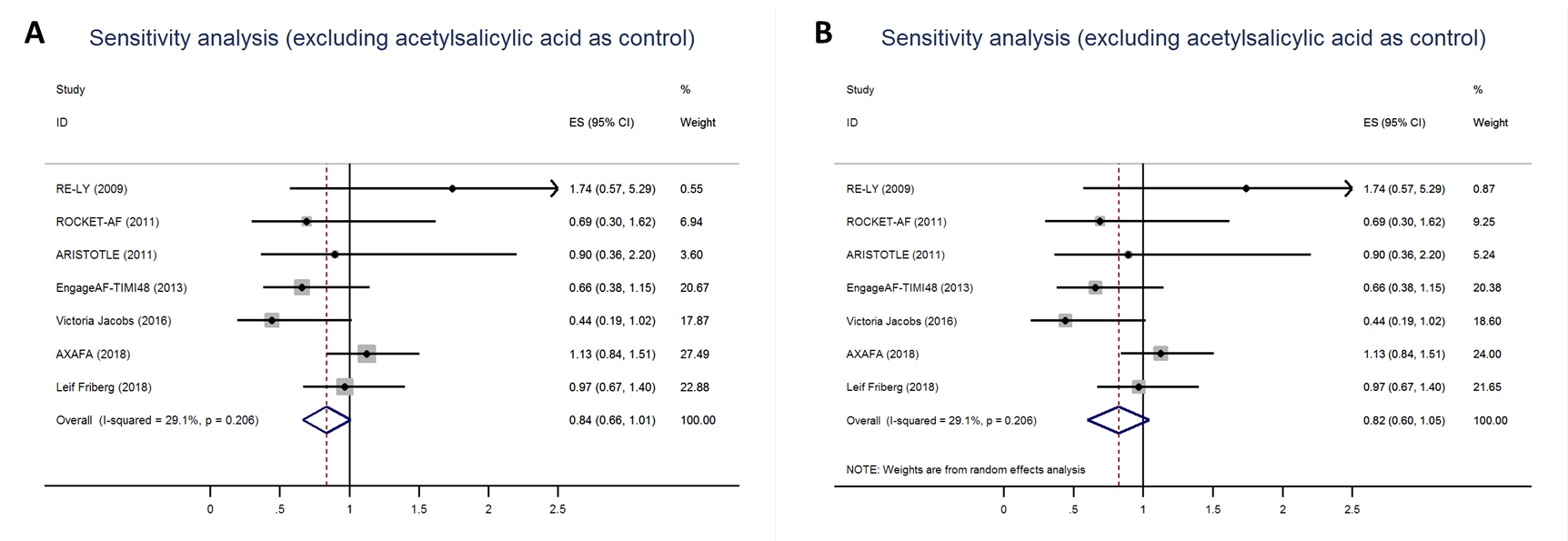
**Figure S7.** Sensitivity analysis by excluding study involved acetylsalicylic acid as control using fixed-effect model (A) and random-effect model (B).


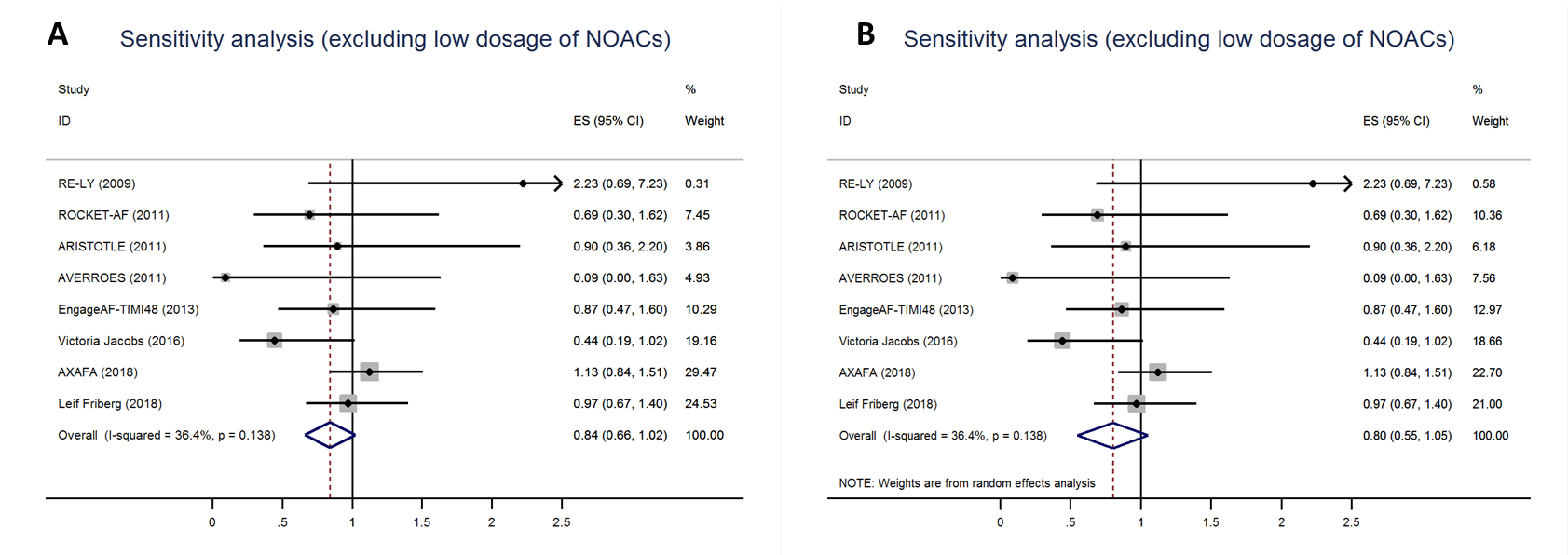
**Figure S8.** Sensitivity analysis by excluding low dosage arms of NOACs using fixed-effect model (A) and random-effect model (B).


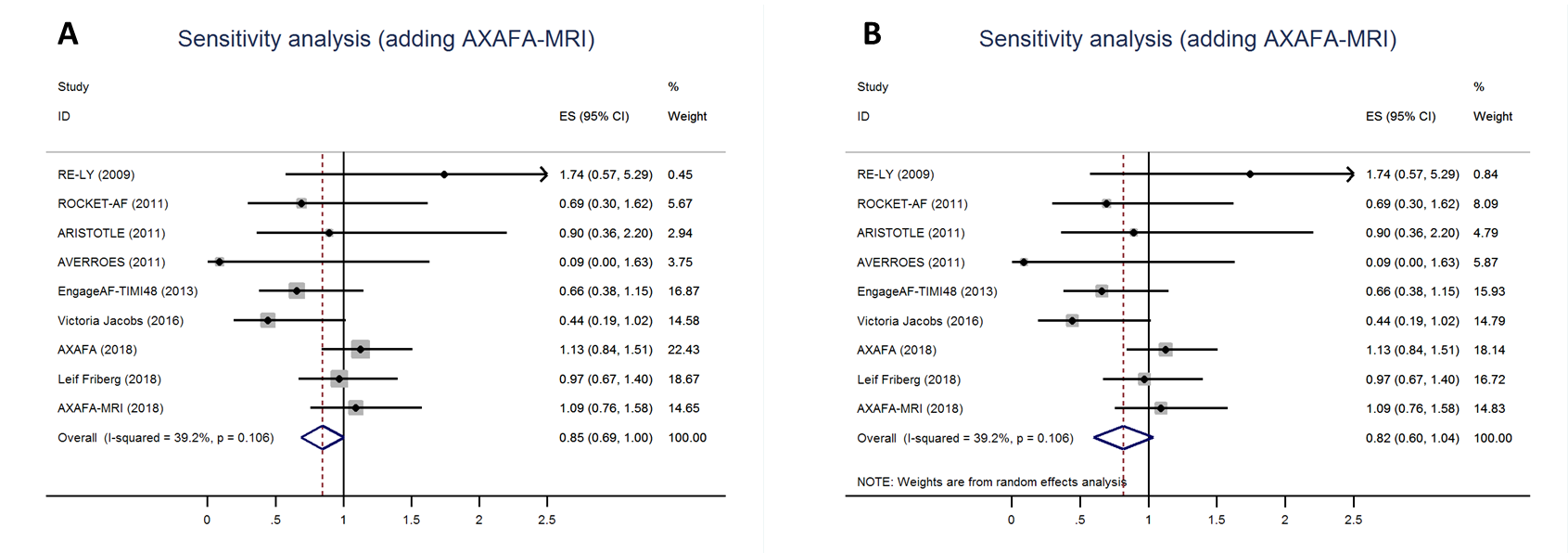


**Figure S9.** Sensitivity analysis by adding the data of magnetic resonance imaging (MRI) sub-study in AXAFA trial using fixed-effect model (A) and random-effect model (B).


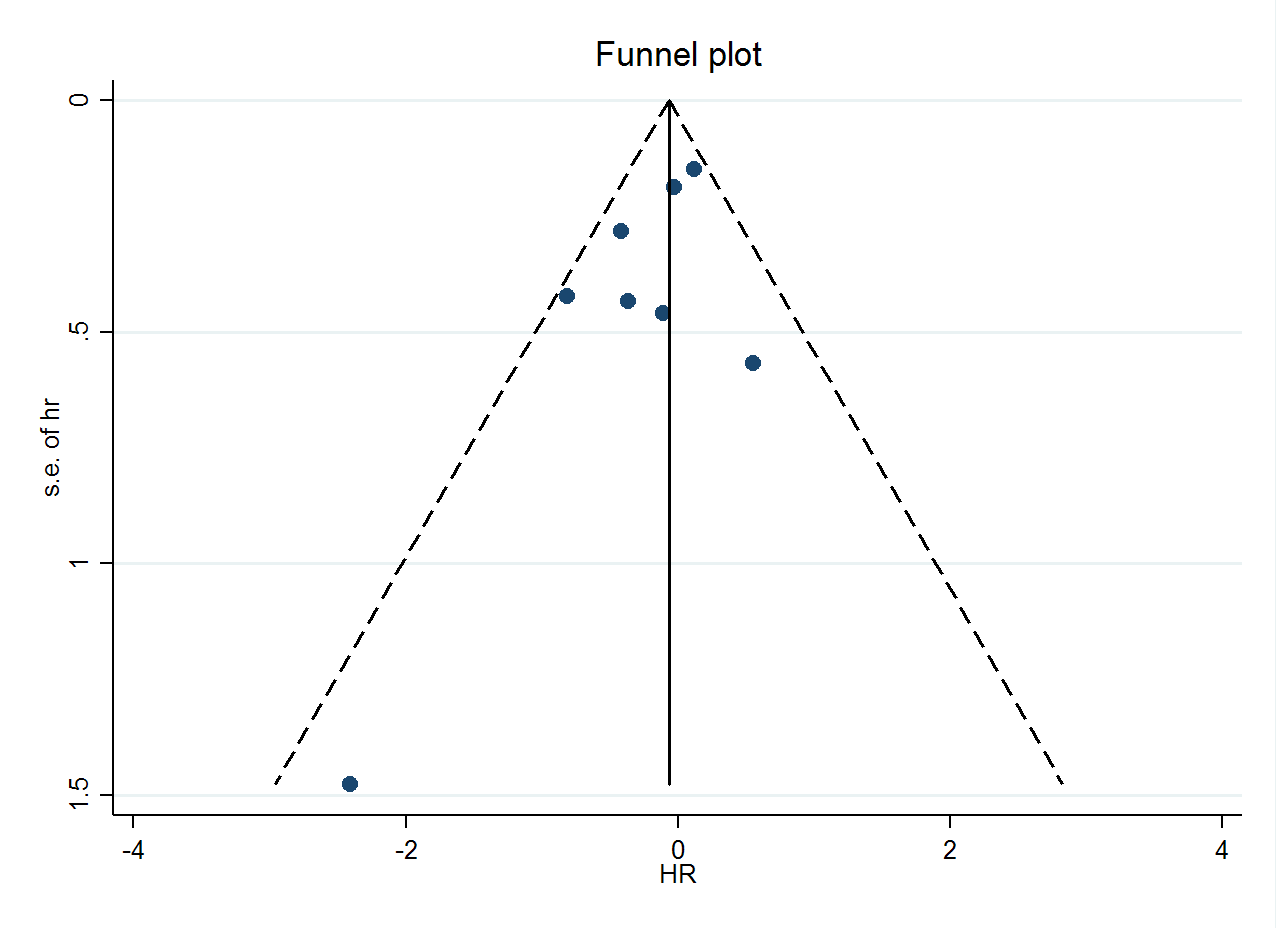
**Figure S10. Funnel plot**

**References**

Abraham, N.S., Singh, S., Alexander, G.C., Heien, H., Haas, L.R., Crown, W., et al. (2015). Comparative risk of gastrointestinal bleeding with dabigatran, rivaroxaban, and warfarin: population based cohort study. *BMJ* 350**,** h1857. doi: 10.1136/bmj.h1857.

Alonso, A., Bengtson, L.G., MacLehose, R.F., Lutsey, P.L., Chen, L.Y., and Lakshminarayan, K. (2014). Intracranial hemorrhage mortality in atrial fibrillation patients treated with dabigatran or warfarin. *Stroke* 45(8)**,** 2286-2291. doi: 10.1161/STROKEAHA.114.006016.

Avgil-Tsadok, M., Jackevicius, C.A., Essebag, V., Eisenberg, M.J., Rahme, E., Behlouli, H., et al. (2016). Dabigatran use in elderly patients with atrial fibrillation. *Thromb Haemost* 115(1)**,** 152-160. doi: 10.1160/TH15-03-0247.

Cha, M.J., Choi, E.K., Han, K.D., Lee, S.R., Lim, W.H., Oh, S., et al. (2017). Effectiveness and Safety of Non-Vitamin K Antagonist Oral Anticoagulants in Asian Patients With Atrial Fibrillation. *Stroke* 48(11)**,** 3040-3048. doi: 10.1161/strokeaha.117.018773.

Chan, Y.H., Kuo, C.T., Yeh, Y.H., Chang, S.H., Wu, L.S., Lee, H.F., et al. (2016). Thromboembolic, Bleeding, and Mortality Risks of Rivaroxaban and Dabigatran in Asians With Nonvalvular Atrial Fibrillation. *J Am Coll Cardiol* 68(13)**,** 1389-1401. doi: 10.1016/j.jacc.2016.06.062.

Chang, H.Y., Zhou, M., Tang, W., Alexander, G.C., and Singh, S. (2015). Risk of gastrointestinal bleeding associated with oral anticoagulants: population based retrospective cohort study. *BMJ* 350**,** h1585. doi: 10.1136/bmj.h1585.

Coleman, C.I., Antz, M., Bowrin, K., Evers, T., Simard, E.P., Bonnemeier, H., et al. (2016). Real-world evidence of stroke prevention in patients with nonvalvular atrial fibrillation in the United States: the REVISIT-US study. *Curr Med Res Opin* 32(12)**,** 2047-2053. doi: 10.1080/03007995.2016.1237937.

Coleman, C.I., Peacock, W.F., Bunz, T.J., and Alberts, M.J. (2017). Effectiveness and Safety of Apixaban, Dabigatran, and Rivaroxaban Versus Warfarin in Patients With Nonvalvular Atrial Fibrillation and Previous Stroke or Transient Ischemic Attack. *Stroke* 48(8)**,** 2142-2149. doi: 10.1161/strokeaha.117.017474.

Connolly, S.J., Eikelboom, J., Joyner, C., Diener, H.C., Hart, R., Golitsyn, S., et al. (2011). Apixaban in patients with atrial fibrillation. *N Engl J Med* 364(9)**,** 806-817. doi: 10.1056/NEJMoa1007432.

Connolly, S.J., Ezekowitz, M.D., Yusuf, S., Eikelboom, J., Oldgren, J., Parekh, A., et al. (2009). Dabigatran versus warfarin in patients with atrial fibrillation. *N Engl J Med* 361(12)**,** 1139-1151. doi: 10.1056/NEJMoa0905561.

Ellis, M.H., Neuman, T., Bitterman, H., Dotan, S.G., Hammerman, A., Battat, E., et al. (2016). Bleeding in patients with atrial fibrillation treated with dabigatran, rivaroxaban or warfarin: A retrospective population-based cohort study. *Eur J Intern Med* 33**,** 55-59. doi: 10.1016/j.ejim.2016.05.023.

Friberg, L., and Oldgren, J. (2017). Efficacy and safety of non-Vitamin K antagonist oral anticoagulants compared with warfarin in patients with atrial fibrillation. *Open Heart* 4(2). doi: 10.1136/openhrt-2017-000682.

Friberg, L., and Rosenqvist, M. (2018). Less dementia with oral anticoagulation in atrial fibrillation. *Eur Heart J* 39(6)**,** 453-460. doi: 10.1093/eurheartj/ehx579.

Gieling, E.M., van den Ham, H.A., van Onzenoort, H., Bos, J., Kramers, C., de Boer, A., et al. (2017). Risk of major bleeding and stroke associated with the use of vitamin K antagonists, nonvitamin K antagonist oral anticoagulants and aspirin in patients with atrial fibrillation: a cohort study. *British Journal of Clinical Pharmacology* 83(8)**,** 1844-1859. doi: 10.1111/bcp.13265.

Giugliano, R.P., Ruff, C.T., Braunwald, E., Murphy, S.A., Wiviott, S.D., Halperin, J.L., et al. (2013). Edoxaban versus warfarin in patients with atrial fibrillation. *N Engl J Med* 369(22)**,** 2093-2104. doi: 10.1056/NEJMoa1310907.

Gorst-Rasmussen, A., Lip, G.Y., and Bjerregaard Larsen, T. (2016). Rivaroxaban versus warfarin and dabigatran in atrial fibrillation: comparative effectiveness and safety in Danish routine care. *Pharmacoepidemiol Drug Saf* 25(11)**,** 1236-1244. doi: 10.1002/pds.4034.

Granger, C.B., Alexander, J.H., McMurray, J.J., Lopes, R.D., Hylek, E.M., Hanna, M., et al. (2011). Apixaban versus warfarin in patients with atrial fibrillation. *N Engl J Med* 365(11)**,** 981-992. doi: 10.1056/NEJMoa1107039.

Halvorsen, S., Ghanima, W., Fride Tvete, I., Hoxmark, C., Falck, P., Solli, O., et al. (2017). A nationwide registry study to compare bleeding rates in patients with atrial fibrillation being prescribed oral anticoagulants. *Eur Heart J Cardiovasc Pharmacother* 3(1)**,** 28-36. doi: 10.1093/ehjcvp/pvw031.

Harel, Z., Mamdani, M., Juurlink, D.N., Garg, A.X., Wald, R., Yao, Z., et al. (2016). Novel Oral Anticoagulants and the Risk of Major Hemorrhage in Elderly Patients With Chronic Kidney Disease: A Nested Case-Control Study. *Can J Cardiol* 32(8)**,** 986.e917-922. doi: 10.1016/j.cjca.2016.01.013.

Hernandez, I., Baik, S.H., Pinera, A., and Zhang, Y. (2015). Risk of bleeding with dabigatran in atrial fibrillation. *JAMA Intern Med* 175(1)**,** 18-24. doi: 10.1001/jamainternmed.2014.5398.

Hernandez, I., and Zhang, Y. (2017). Comparing Stroke and Bleeding with Rivaroxaban and Dabigatran in Atrial Fibrillation: Analysis of the US Medicare Part D Data. *Am J Cardiovasc Drugs* 17(1)**,** 37-47. doi: 10.1007/s40256-016-0189-9.

Hernandez, I., Zhang, Y., Brooks, M.M., Chin, P.K., and Saba, S. (2017a). Anticoagulation Use and Clinical Outcomes After Major Bleeding on Dabigatran or Warfarin in Atrial Fibrillation. *Stroke* 48(1)**,** 159-166. doi: 10.1161/strokeaha.116.015150.

Hernandez, I., Zhang, Y., and Saba, S. (2017b). Comparison of the Effectiveness and Safety of Apixaban, Dabigatran, Rivaroxaban, and Warfarin in Newly Diagnosed Atrial Fibrillation. *Am J Cardiol* 120(10)**,** 1813-1819. doi: 10.1016/j.amjcard.2017.07.092.

Ho, C.W., Ho, M.H., Chan, P.H., Hai, J.J., Cheung, E., Yeung, C.Y., et al. (2015). Ischemic stroke and intracranial hemorrhage with aspirin, dabigatran, and warfarin: impact of quality of anticoagulation control. *Stroke* 46(1)**,** 23-30. doi: 10.1161/STROKEAHA.114.006476.

Hohnloser, S.H., Basic, E., and Nabauer, M. (2017). Comparative risk of major bleeding with new oral anticoagulants (NOACs) and phenprocoumon in patients with atrial fibrillation: a post-marketing surveillance study. *Clin Res Cardiol* 106(8)**,** 618-628. doi: 10.1007/s00392-017-1098-x.

Jacobs, V., May, H.T., Bair, T.L., Crandall, B.G., Cutler, M.J., Day, J.D., et al. (2016). Long-Term Population-Based Cerebral Ischemic Event and Cognitive Outcomes of Direct Oral Anticoagulants Compared With Warfarin Among Long-term Anticoagulated Patients for Atrial Fibrillation. *Am J Cardiol* 118(2)**,** 210-214. doi: 10.1016/j.amjcard.2016.04.039.

Kirchhof, P., Haeusler, K.G., Blank, B., De Bono, J., Callans, D., Elvan, A., et al. (2018). Apixaban in patients at risk of stroke undergoing atrial fibrillation ablation. *Eur Heart J*. doi: 10.1093/eurheartj/ehy176.

Kohsaka, S., Murata, T., Izumi, N., Katada, J., Wang, F., and Terayama, Y. (2017). Bleeding risk of apixaban, dabigatran, and low-dose rivaroxaban compared with warfarin in Japanese patients with non-valvular atrial fibrillation: a propensity matched analysis of administrative claims data. *Curr Med Res Opin* 33(11)**,** 1955-1963. doi: 10.1080/03007995.2017.1374935.

Korenstra, J., Wijtvliet, E.P., Veeger, N.J., Geluk, C.A., Bartels, G.L., Posma, J.L., et al. (2016). Effectiveness and safety of dabigatran versus acenocoumarol in 'real-world' patients with atrial fibrillation. *Europace* 18(9)**,** 1319-1327. doi: 10.1093/europace/euv397.

Lamberts, M., Staerk, L., Olesen, J.B., Fosbol, E.L., Hansen, M.L., Harboe, L., et al. (2017). Major Bleeding Complications and Persistence With Oral Anticoagulation in Non-Valvular Atrial Fibrillation: Contemporary Findings in Real-Life Danish Patients. *J Am Heart Assoc* 6(2). doi: 10.1161/jaha.116.004517.

Larsen, T.B., Gorst-Rasmussen, A., Rasmussen, L.H., Skjoth, F., Rosenzweig, M., and Lip, G.Y. (2014a). Bleeding events among new starters and switchers to dabigatran compared with warfarin in atrial fibrillation. *Am J Med* 127(7)**,** 650-656 e655. doi: 10.1016/j.amjmed.2014.01.031.

Larsen, T.B., Rasmussen, L.H., Gorst-Rasmussen, A., Skjoth, F., Lane, D.A., and Lip, G.Y. (2014b). Dabigatran and warfarin for secondary prevention of stroke in atrial fibrillation patients: a nationwide cohort study. *Am J Med* 127(12)**,** 1172-1178 e1175. doi: 10.1016/j.amjmed.2014.07.023.

Larsen, T.B., Rasmussen, L.H., Skjoth, F., Due, K.M., Callreus, T., Rosenzweig, M., et al. (2013). Efficacy and safety of dabigatran etexilate and warfarin in "real-world" patients with atrial fibrillation: a prospective nationwide cohort study. *J Am Coll Cardiol* 61(22)**,** 2264-2273. doi: 10.1016/j.jacc.2013.03.020.

Larsen, T.B., Skjoth, F., Nielsen, P.B., Kjaeldgaard, J.N., and Lip, G.Y. (2016). Comparative effectiveness and safety of non-vitamin K antagonist oral anticoagulants and warfarin in patients with atrial fibrillation: propensity weighted nationwide cohort study. *BMJ* 353**,** i3189. doi: 10.1136/bmj.i3189.

Lauffenburger, J.C., Farley, J.F., Gehi, A.K., Rhoney, D.H., Brookhart, M.A., and Fang, G. (2015). Effectiveness and safety of dabigatran and warfarin in real-world US patients with non-valvular atrial fibrillation: a retrospective cohort study. *J Am Heart Assoc* 4(4). doi: 10.1161/JAHA.115.001798.

Li, W.H., Huang, D., Chiang, C.E., Lau, C.P., Tse, H.F., Chan, E.W., et al. (2017). Efficacy and safety of dabigatran, rivaroxaban, and warfarin for stroke prevention in Chinese patients with atrial fibrillation: the Hong Kong Atrial Fibrillation Project. *Clin Cardiol* 40(4)**,** 222-229. doi: 10.1002/clc.22649.

Lin, J., Trocio, J., Gupta, K., Mardekian, J., Lingohr-Smith, M., Menges, B., et al. (2017). Major bleeding risk and healthcare economic outcomes of non-valvular atrial fibrillation patients newly-initiated with oral anticoagulant therapy in the real-world setting. *J Med Econ* 20(9)**,** 952-961. doi: 10.1080/13696998.2017.1341902.

Lip, G.Y., Keshishian, A., Kamble, S., Pan, X., Mardekian, J., Horblyuk, R., et al. (2016a). Real-world comparison of major bleeding risk among non-valvular atrial fibrillation patients initiated on apixaban, dabigatran, rivaroxaban, or warfarin. A propensity score matched analysis. *Thromb Haemost* 116(5)**,** 975-986. doi: 10.1160/TH16-05-0403.

Lip, G.Y., Pan, X., Kamble, S., Kawabata, H., Mardekian, J., Masseria, C., et al. (2016b). Major bleeding risk among non-valvular atrial fibrillation patients initiated on apixaban, dabigatran, rivaroxaban or warfarin: a "real-world" observational study in the United States. *Int J Clin Pract* 70(9)**,** 752-763. doi: 10.1111/ijcp.12863.

Lip, G.Y.H., Skjoth, F., Nielsen, P.B., Kjaeldgaard, J.N., and Larsen, T.B. (2017). Effectiveness and Safety of Standard-Dose Nonvitamin K Antagonist Oral Anticoagulants and Warfarin Among Patients With Atrial Fibrillation With a Single Stroke Risk Factor: A Nationwide Cohort Study. *JAMA Cardiol* 2(8)**,** 872-881. doi: 10.1001/jamacardio.2017.1883.

Maura, G., Blotiere, P.O., Bouillon, K., Billionnet, C., Ricordeau, P., Alla, F., et al. (2015). Comparison of the short-term risk of bleeding and arterial thromboembolic events in nonvalvular atrial fibrillation patients newly treated with dabigatran or rivaroxaban versus vitamin K antagonists: a French nationwide propensity-matched cohort study. *Circulation* 132(13)**,** 1252-1260. doi: 10.1161/CIRCULATIONAHA.115.015710.

Naganuma, M., Shiga, T., Nagao, T., Suzuki, A., Murasaki, K., and Hagiwara, N. (2017). Effectiveness and safety of dabigatran versus warfarin in "real-world" Japanese patients with atrial fibrillation: A single-center observational study. *J Arrhythm* 33(2)**,** 107-110. doi: 10.1016/j.joa.2016.07.011

10.1016/j.joa.2016.07.007.

Nielsen, P.B., Skjoth, F., Sogaard, M., Kjaeldgaard, J.N., Lip, G.Y., and Larsen, T.B. (2017). Effectiveness and safety of reduced dose non-vitamin K antagonist oral anticoagulants and warfarin in patients with atrial fibrillation: propensity weighted nationwide cohort study. *Bmj* 356**,** j510. doi: 10.1136/bmj.j510.

Nishtala, P.S., Gnjidic, D., Jamieson, H.A., Hanger, H.C., Kaluarachchi, C., and Hilmer, S.N. (2016). 'Real-world' haemorrhagic rates for warfarin and dabigatran using population-level data in New Zealand. *Int J Cardiol* 203**,** 746-752. doi: 10.1016/j.ijcard.2015.11.067.

Norby, F.L., Bengtson, L.G.S., Lutsey, P.L., Chen, L.Y., MacLehose, R.F., Chamberlain, A.M., et al. (2017). Comparative effectiveness of rivaroxaban versus warfarin or dabigatran for the treatment of patients with non-valvular atrial fibrillation. *BMC Cardiovasc Disord* 17(1)**,** 238. doi: 10.1186/s12872-017-0672-5.

Palamaner Subash Shantha, G., Bhave, P.D., Girotra, S., Hodgson-Zingman, D., Mazur, A., Giudici, M., et al. (2017). Sex-Specific Comparative Effectiveness of Oral Anticoagulants in Elderly Patients With Newly Diagnosed Atrial Fibrillation. *Circ Cardiovasc Qual Outcomes* 10(4). doi: 10.1097/aln.0000000000001631

10.1161/circoutcomes.116.003418.

Patel, M.R., Mahaffey, K.W., Garg, J., Pan, G., Singer, D.E., Hacke, W., et al. (2011). Rivaroxaban versus warfarin in nonvalvular atrial fibrillation. *N Engl J Med* 365(10)**,** 883-891. doi: 10.1056/NEJMoa1009638.

Song, X., Gandhi, P., Gilligan, A.M., Arora, P., Wang, C., Henriques, C., et al. (2017). Comparison of all-cause, stroke, and bleed-specific healthcare resource utilization among patients with non-valvular atrial fibrillation (NVAF) and newly treated with dabigatran or warfarin. *Expert Rev Pharmacoecon Outcomes Res***,** 1-10. doi: 10.1080/14737167.2017.1347041.

Sorensen, R., Gislason, G., Torp-Pedersen, C., Olesen, J.B., Fosbol, E.L., Hvidtfeldt, M.W., et al. (2013). Dabigatran use in Danish atrial fibrillation patients in 2011: a nationwide study. *BMJ Open* 3(5). doi: 10.1136/bmjopen-2013-002758.

Staerk, L., Fosbol, E.L., Lip, G.Y.H., Lamberts, M., Bonde, A.N., Torp-Pedersen, C., et al. (2017a). Ischaemic and haemorrhagic stroke associated with non-vitamin K antagonist oral anticoagulants and warfarin use in patients with atrial fibrillation: a nationwide cohort study. *Eur Heart J* 38(12)**,** 907-915. doi: 10.1093/eurheartj/ehw496.

Staerk, L., Gerds, T.A., Lip, G.Y.H., Ozenne, B., Bonde, A.N., Lamberts, M., et al. (2017b). Standard and reduced doses of dabigatran, rivaroxaban and apixaban for stroke prevention in atrial fibrillation: a nationwide cohort study. *J Intern Med*. doi: 10.1111/joim.12683.

Staerk, L., Gislason, G.H., Lip, G.Y., Fosbol, E.L., Hansen, M.L., Lamberts, M., et al. (2015). Risk of gastrointestinal adverse effects of dabigatran compared with warfarin among patients with atrial fibrillation: a nationwide cohort study. *Europace* 17(8)**,** 1215-1222. doi: 10.1093/europace/euv119.

Stolk, L.M., de Vries, F., Ebbelaar, C., de Boer, A., Schalekamp, T., Souverein, P., et al. (2017). Risk of myocardial infarction in patients with atrial fibrillation using vitamin K antagonists, aspirin or direct acting oral anticoagulants. *Br J Clin Pharmacol* 83(8)**,** 1835-1843. doi: 10.1111/bcp.13264.

Vaughan Sarrazin, M.S., Jones, M., Mazur, A., Chrischilles, E., and Cram, P. (2014). Bleeding rates in Veterans Affairs patients with atrial fibrillation who switch from warfarin to dabigatran. *Am J Med* 127(12)**,** 1179-1185. doi: 10.1016/j.amjmed.2014.07.024.

Yamashita, Y., Uozumi, R., Hamatani, Y., Esato, M., Chun, Y.H., Tsuji, H., et al. (2017). Current status and outcomes of direct oral anticoagulant use in real-world atrial fibrillation patients ― fushimi AF registry ―. *Circulation Journal* 81(9)**,** 1278-1285. doi: 10.1253/circj.CJ-16-1337.

Yao, X., Abraham, N.S., Sangaralingham, L.R., Bellolio, M.F., McBane, R.D., Shah, N.D., et al. (2016). Effectiveness and Safety of Dabigatran, Rivaroxaban, and Apixaban Versus Warfarin in Nonvalvular Atrial Fibrillation. *J Am Heart Assoc* 5(6). doi: 10.1161/JAHA.116.003725.

Yap, L.B., Eng, D.T., Sivalingam, L., Rusani, B.I., Umadevan, D., Muhammad, Z., et al. (2016). A Comparison of Dabigatran With Warfarin for Stroke Prevention in Atrial Fibrillation in an Asian Population. *Clin Appl Thromb Hemost* 22(8)**,** 792-797. doi: 10.1177/1076029615584664.

Yavuz, B., Ayturk, M., Ozkan, S., Ozturk, M., Topaloglu, C., Aksoy, H., et al. (2016). A real world data of dabigatran etexilate: multicenter registry of oral anticoagulants in nonvalvular atrial fibrillation. *J Thromb Thrombolysis* 42(3)**,** 399-404. doi: 10.1007/s11239-016-1361-4.
